# Central Topography of Cranial Motor Nuclei Controlled by Differential Cadherin Expression

**DOI:** 10.1016/j.cub.2014.08.067

**Published:** 2014-11-03

**Authors:** Marc Astick, Kristina Tubby, Waleed M. Mubarak, Sarah Guthrie, Stephen R. Price

**Affiliations:** 1 Research Department of Cell and Developmental Biology, University College London, Gower Street, London WC1E 6BT, UK; 2 MRC Centre for Developmental Neurobiology, 4^th^ Floor New Hunt’s House, Kings College, Guy’s Campus, London SE1 1UL, UK

## Abstract

Neuronal nuclei are prominent, evolutionarily conserved features of vertebrate central nervous system (CNS) organization [1]. Nuclei are clusters of soma of functionally related neurons and are located in highly stereotyped positions. Establishment of this CNS topography is critical to neural circuit assembly. However, little is known of either the cellular or molecular mechanisms that drive nucleus formation during development, a process termed nucleogenesis [2, 3, 4, 5]. Brainstem motor neurons, which contribute axons to distinct cranial nerves and whose functions are essential to vertebrate survival, are organized exclusively as nuclei. Cranial motor nuclei are composed of two main classes, termed branchiomotor/visceromotor and somatomotor [6]. Each of these classes innervates evolutionarily distinct structures, for example, the branchial arches and eyes, respectively. Additionally, each class is generated by distinct progenitor cell populations and is defined by differential transcription factor expression [7, 8]; for example, Hb9 distinguishes somatomotor from branchiomotor neurons. We characterized the time course of cranial motornucleogenesis, finding that despite differences in cellular origin, segregation of branchiomotor and somatomotor nuclei occurs actively, passing through a phase of each being intermingled. We also found that differential expression of cadherin cell adhesion family members uniquely defines each motor nucleus. We show that cadherin expression is critical to nucleogenesis as its perturbation degrades nucleus topography predictably.

## Highlights

• Cranial motor nucleogenesis occurs through an active process of segregation
• Differential cadherin expression defines cranial motor nuclei
• Cadherin expression drives specificity of cranial motor nucleus segregation
• Cadherin expression does not affect cranial motor neuron migration

## Teaser

Astick et al. show that differential cadherin cell adhesion molecule expression defines brainstem motor neurons during their coalescence into discrete motor nuclei. No two motor nuclei express the same combination of cadherins. They demonstrate that the specificity of this expression is critical to the segregation of brainstem motor nuclei.

## Results and Discussion

To investigate the mechanisms of somatomotor versus branchiomotor nucleogenesis, we focused our attention on rhombomere 5 (r5) and r8 of the brainstem of the chicken embryo. Cranial motor neurons at these levels are born within these rhombomeres, and there is little rostrocaudal migration of the motor neurons while they take up their stereotyped positions. In all, eight distinct motor nuclei are generated at r5 and r8, and these motor neurons contribute axons to five cranial nerves. Four distinct motor nuclei, two somatic and two branchiomotor, are located in r5. There, the somatic abducens and accessory abducens nuclei project axons via the VI^th^ cranial nerve to the lateral rectus muscle and retractor bulbi muscles of the eye [9, 10], respectively. The branchiomotor dorsal and ventral divisions of the facial motor nucleus [11] contribute axons to the VII^th^ cranial nerve and control beak opening (dorsal nucleus) or innervate suprahyoid muscles of the tongue (ventral nucleus) [6, 12]. At r8, the glossopharyngeal (IX^th^) and vagal (X^th^) (both branchio-/visceromotor) and hypoglossal (XII^th^) (somatomotor) cranial nerves receive axons from four spatially segregated clusters of motor neurons.

### Cranial Motor Nucleogenesis Requires an Active Segregation of the Motor Nuclei

**Figure 1:**
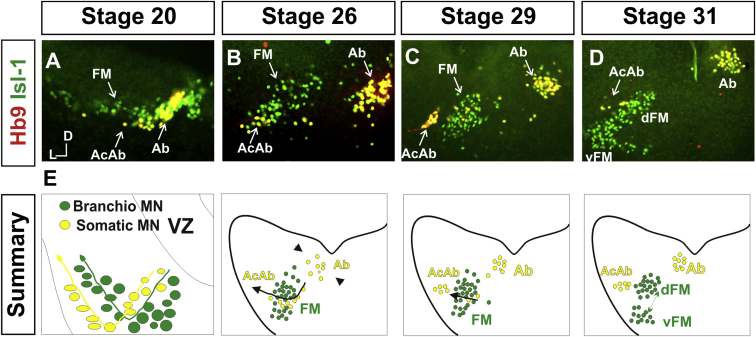
Development of Motor Nucleus Formation at Rhombomere 5 (A–D) Branchiomotor (Hb9^−^/Islet-1^+^) and Somatomotor (Hb9^+^Islet-1^+^) neurons in r5 at st20 (A), st26 (B), st29 (C), and st31 (D). Arrows show accessory abducens (AcAb), abducens (Ab) or facial motorneurons. Abducens neurons form initially a relatively undefined cluster (A and B) that becomes more coherent by st31 (D). Accessory abducens cells and facial neurons have distinct migration paths which converge and then segregate and cluster by st31. (E) Summary. VZ is ventricular zone, FM is facial motor neurons, and d and v are dorsal and ventral, respectively. See also Figure S1.

We first characterized the time course of cranial motor nucleogenesis at r5 by analyzing immunofluorescence for Hb9^+^ and Islet-1^+^ somatomotor neurons (SMNs) and Hb9^−^, Islet-1^+^ branchiomotor neurons (BMNs) (Figure 1). We found that the early trajectories of the migration of facial and accessory abducens neurons were different with adjacent paths radially followed by distinct paths dorsally (Figure 1A) [13]. However, at stage 26 (st26) [14], the migratory streams of both the presumptive facial and accessory abducens nuclei have converged and the neurons of each nucleus are intermingled (Figure 1B). By st31, the accessory abducens has segregated from the facial nucleus, residing dorsolaterally to it, and the loose aggregate of the facial nucleus has separated into dorsal and ventral subdivisions (Figures 1C–1E). In contrast, the abducens neurons migrated only radially and formed a loose aggregate of cells close to the midline, which became better defined between st26 and st31 (Figure 1).

A similar scheme of motor nucleogenesis was observed at r8 (Figure S1 available online). SMNs and BMNs are generated in adjacent progenitor domains (Figure S1A). However, at st26, considerable mixing of the somatomotor XII^th^ neurons with the branchiomotor/visceromotor neurons is observed (Figure S1B). The four distinct groupings of IX^th^, X^th^ and dorsal and ventral divisions of the XII^th^ nuclei at r8 emerge by st29 to st32 (Figures S1C and S1D).

Thus, at both r5 and r8, characteristic migratory streams of motor neurons resulted in initially scattered and intermingled cell groups that segregated in a highly stereotyped manner. Despite distinctions in birthplace and initial paths of migration, branchiomotor and somatomotor neurons pass through a phase of being mixed and then sort out from one another. Additionally, both somatic and branchiomotor nuclei initially form loosely defined nuclei and coalesce into characteristic, distinct locations within the brainstem. Thus, cranial motor nucleogenesis involves an active process of segregation of the nuclei, despite early differences in progenitor cell location. We next asked how the specificity by which cranial motor nuclei sort from one another could be driven molecularly.

### Differential Cadherin Expression Defines Cranial Motor Nuclei

**Figure 2:**
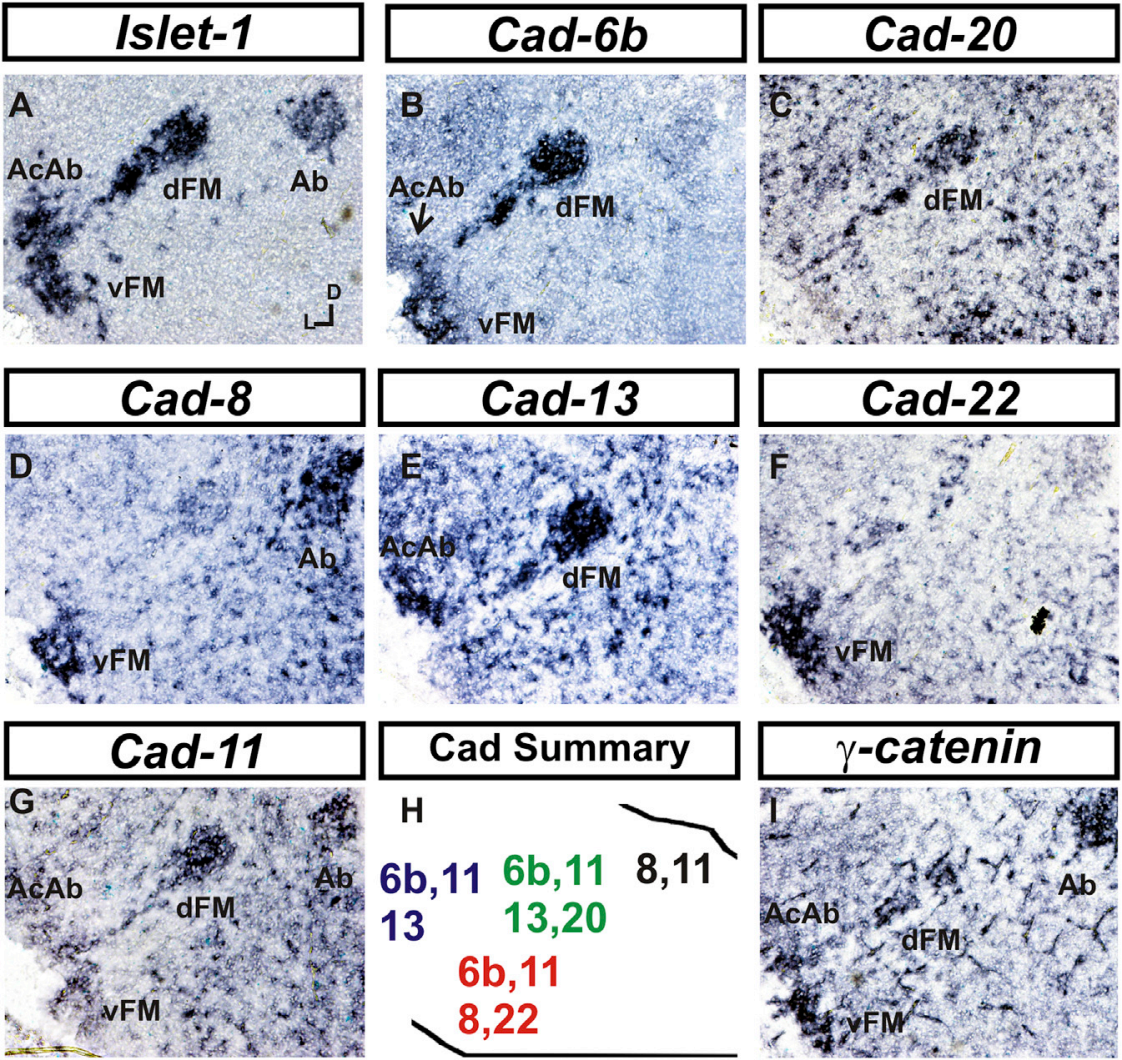
Cadherin and Catenin Expression in r5 at stage 35 (A) *Islet-1* in situ hybridization shows the topography of nucleus positioning of abducens (Ab), accessory abducens (AcAb), dorsal facial nucleus (dFM), and ventral facial motor nucleus (vFM). (B–H) *cad-6b* (B), *cad–20* (C), *cad-8* (D), *cad-13* (E), *cad–22* (F), and *cad-11* (G) expression on adjacent sections. A summary of differential cadherin expression in r5 motor nuclei is shown in (H). Note that no two nuclei share the same cadherin combination and that the accessory abducens and dorsal facial nuclei differ by the expression of *cad-20* in the dorsal facial nucleus. (I) *γ-catenin* expression in all four motor nuclei at r5. See also Figure S2.

We focused our attention on the cadherin family of cell adhesion molecules as candidates to drive the sorting and segregation of cranial motor nuclei, as they play key roles in the organization of spinal lateral motor column neurons [15, 16, 17, 18, 19, 20, 21]. We surveyed classical cadherin expression at st35 and found differential expression of six cadherins (*cadherin-6b* [22], *cadherin-8*, *cadherin-11*, *cadherin-13*, *cadherin-20*, and *cadherin-22*) in the cranial motor nuclei of r5 and r8 (Figures 2A–2I and S1E–S1P). For example, at r5, all four nuclei expressed *cadherin-11*, whereas three nuclei expressed *cadherin-6b* and two nuclei expressed *cadherin-8* and/or *cadherin-13*. *cadherin-22* was expressed in only the ventral facial nucleus, and *cadherin-20* was expressed in only the dorsal facial nucleus. Overall, each nucleus was defined by a unique combination of cadherins (Figure 2H). At r8, each of the four distinct groupings of motor neurons also expressed different combinations of cadherins (Figure S1; summarized in Figure S1P). Notably, based on dual immunohistochemistry for Hb9 expression with cadherin in situ hybridization, we were able to distinguish two subsets of the ventral hypoglossal motor nucleus on the basis of the differential expression of *cadherin-13* and *cadherin-6b*, with *cadherin-13* being predominantly expressed in a lateral grouping of the ventral hypoglossal from st29 (Figures S1E–S1G). These two subsets of the ventral hypoglossal become more separated by st36 (Figure S1H), indicating that cadherin expression can delineate nuclei prior to their becoming physically distinguishable clusters of cells. Based on these data, no two cranial motor nuclei at either r5 or r8 shared the same cadherin expression profile (Figures 2H and S1P).

Importantly, *γ-catenin*, an armadillo family member required for cadherin function, was expressed in all motor nuclei at r5 and r8 (Figures 2I and S1I). However, we only observed expression of the related *β-catenin* within the medial subset of the ventral hypoglossal nucleus at r8. This suggests that the major cytoplasmic partner of classical cadherins in cranial motor neurons is γ-catenin and not β-catenin as is commonly assumed.

### Cadherin Expression in Cranial Motor Nuclei Is Highly Dynamic during Development

**Figure 3:**
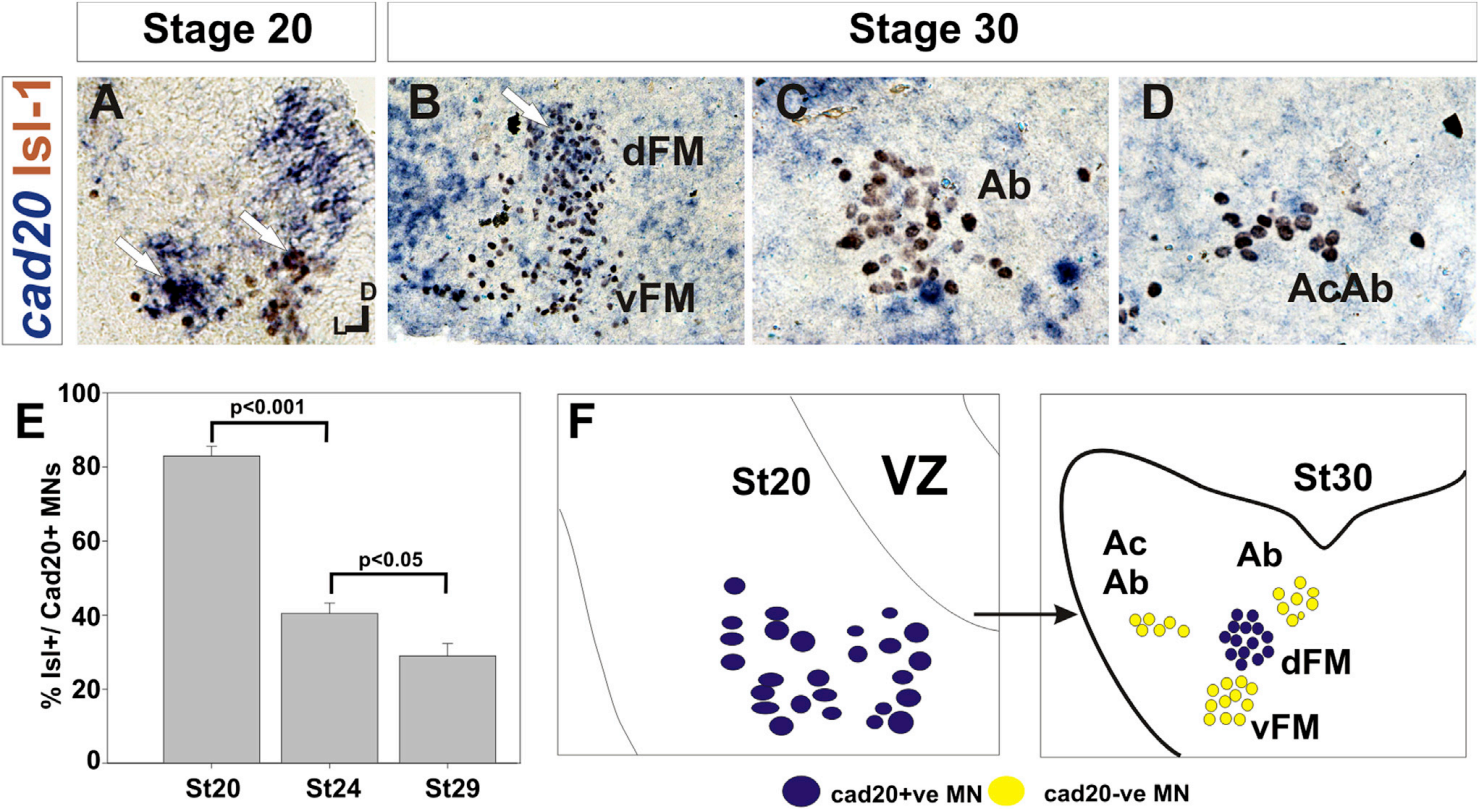
Developmental Time Course of *cadherin-20* Expression at r5 (A–D) *cadherin-20* in situ hybridization with islet-1 immunohistochemistry expression at st20 (A) and st30 (B–D). For clarity, higher-magnification images of facial (B), abducens (C), and accessory abducens (D) nuclei are shown. Arrows indicate Cadherin 20^+^ islet-1^+^ neurons as examples. (E) Quantification of the percentage of islet-1 motor neurons at r5 that express *cadherin-20* at st20, st24, and st29. n = 4 embryos at each stage. Student’s t test p values shown above the bar graphs. Error bars indicate the SEM. (F) Summary of this expression. *cadherin-20* is expressed in the majority of motor neurons at r5 at st20, and this expression is refined to the mature pattern by st30. VZ, ventricular zone. See also Figure S3.

We next asked whether the developmental profile of cadherin expression was consistent with a role in cranial motor nucleogenesis. We assayed cadherin expression at r5 and r8 by double in situ hybridization with islet-1 or Hb9 immunohistochemistry between stages 20 and 30. (Figures 3 and S2). At r5, *cadherin-20* expression was initiated in the majority of motor neurons soon after they were born and was refined from st25 to st30, being downregulated in all neurons other than the dorsal facial nucleus, resulting in the mature pattern of expression (Figures 3A–3D; quantified in Figure 3E and summarized in Figure 3F). In contrast, at r8, *cadherin-20* expression was initiated in a restricted subset of motor neurons, this being maintained through the period of nucleogenesis (Figures S2A–S2C). *cadherin-6b* was expressed in the presumptive ventral hypoglossal at st24 but only became expressed in the dorsal hypoglossal by st27. In contrast, *cadherin-13* expression was not apparent in the hypoglossal nuclei until st29 (Figures S2D–S2H and S1F; summarized in Figures S1G and S2H). Thus, cadherin expression is highly dynamic within cranial motor neurons, and its refinement to a mature pattern coincides with the period of nucleogenesis. Cadherins are thus good candidates to drive cranial motor nucleogenesis.

### Cadherin Function Drives Nucleus Coalescence

**Figure 4:**
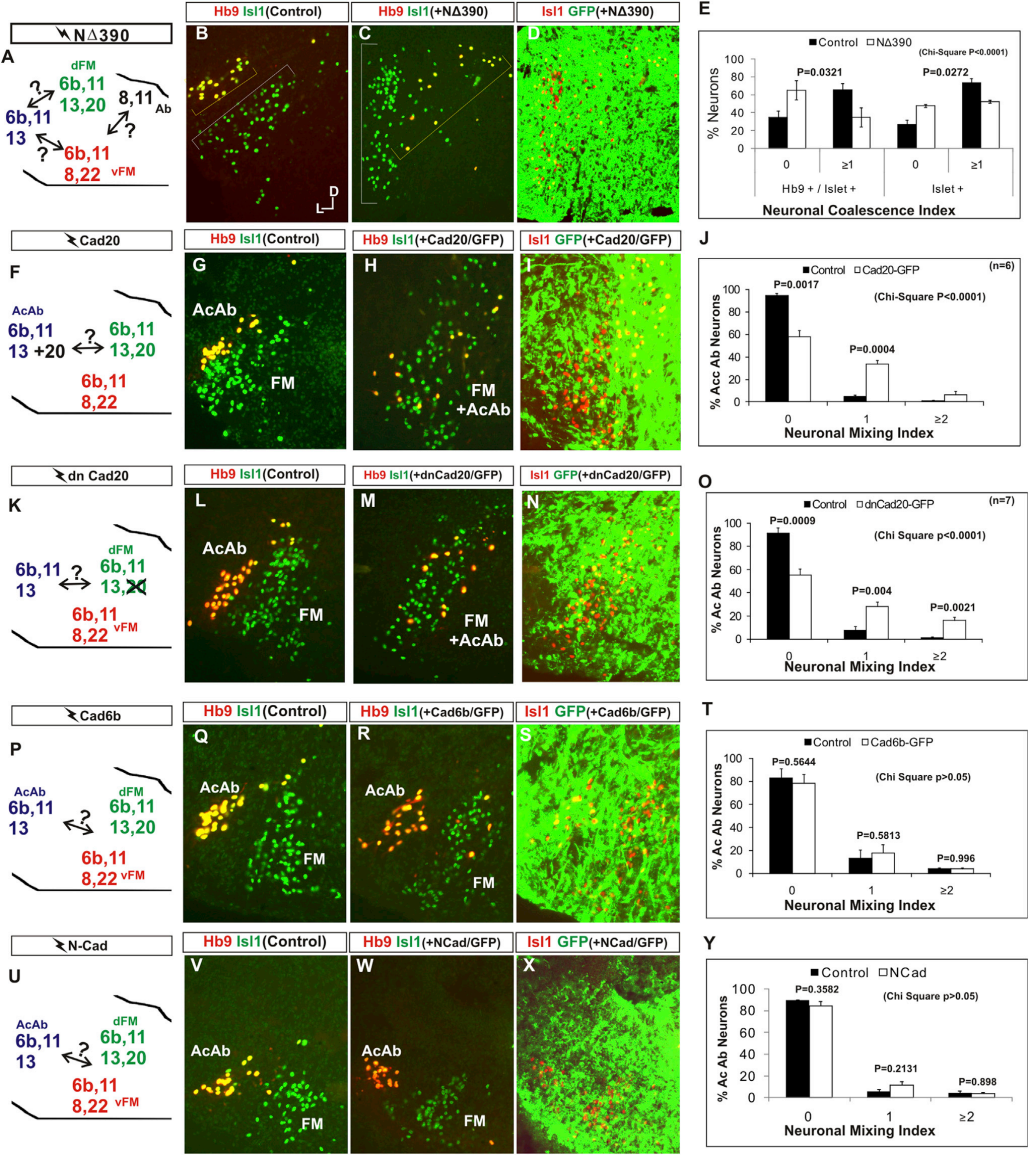
Manipulation of Cadherin Gene Function or Expression Perturbs Cranial Motor Nucleus Topography at r5 (A–E) NΔ390-GFP expression disrupts nucleus clustering at r5 as assayed by Hb9 (B and C) and islet-1 (B and D) immunoreactivity. (A) shows a schematic of the experiment, (B) shows the internal control side of the brainstem, (C) and (D) show the results of NΔ390 expression, and (E) shows quantification of nucleus coalescence using a nucleus coalescence index (see the Supplemental Experimental Procedures). The brackets in (B) and (C) show the spatial extent of facial (FM) and abducens (Ab) nuclei in the control and experimental sides. (F–J) cad-20/GFP coexpression results in the mixing of accessory abducens (AcAb) and facial motor (FM) nuclei assayed by Hb9 (G and H) and islet-1 (G–I) immunoreactivity. (F) shows a schematic of the experiment, (G) shows internal control, and (H) and (I) show results of cad-20 expression, marked by GFP in (I). Quantification of the nucleus mixing (see Supplemental Experimental Procedures) is shown in (J). p values for a Student’s t test of each bin are shown above the graph bars. The chi-squared p value for the entire distribution is p < 0.0001, with two degrees of freedom. (K–O) DNcad-20/GFP coexpression results in a similar mixing of nuclei to cad-20 expression. (K) shows a schematic of the experiment, (L) shows the internal control, and (M) and (N) show the effect of DNcad-20 expression assessed by Hb9 (L and M) and islet-1 (L and N) expression. Electroporation is marked by GFP immunofluorescence in (N). Quantification of nucleus mixing is shown in (O). p values for a Student’s t test of each bin is shown above the graph bars. The chi-squared p value for the entire distribution is p < 0.0001, with two degrees of freedom. (P–T) Cadherin-6b (whose expression is found in both accessory abducens and facial motor nuclei) has no effect on AcAb and FMN segregation at st30 when misexpressed. (P) shows a summary of experiment, (Q) shows the control side of the brainstem, and (R) and (S) show the experimental side of the brainstem. Electroporation is marked by GFP in (S). Quantification of neuronal mixing index is shown in (T). (U–Y) N-cadherin (whose expression is found in neither accessory abducens and facial motor nuclei) has no effect on AcAb and FMN segregation at st30 when misexpressed. (U) shows a summary of experiment, (V) shows the control side of the brainstem, and (W) and (X) show the experimental side of the brainstem. Electroporation is marked by GFP in (X). Quantification of neuronal mixing index is shown in (Y). Error bars indicate the SEM. See also Figure S4.

To test whether cadherin expression controls cranial motor nucleogenesis, we perturbed general cadherin function through the expression of the dominant negative isoform NΔ390 (Figures 4A–4E) Expression was confirmed by GFP immunofluorescence driven by the electroporated plasmid [23, 24, 25, 26] (Figure 4D). After NΔ390 expression, the progenitor domains from which motor neurons arise appeared to be unaffected at either r5 or r8 (Figures S3A–S3F). Consistent with this, the total number of motor neurons was not affected (Supplemental Experimental Procedures and Figure S3G), and motor axons projected out of the brainstem normally (Figure S3H and data not shown). This suggests that cadherin perturbation does not affect motor neuron differentiation and allowed us to assess the effect of NΔ390 expression on motor nucleus formation. We observed a failure of all motor nuclei at r5 and r8 to coalesce after NΔ390 expression (Figures 4A–4E and S3I–S3Q). For example, the accessory abducens and facial motor nuclei at r5 were scattered over a larger area after NΔ390 expression compared to the control (see the yellow brackets in Figures 4B and 4C). This effect was not observed where motor neurons were nonelectroporated (Figures S3I and S3J), suggesting that cell-autonomous cadherin function is essential for nucleogenesis. Taken together, these results suggest that the observed phenotype is motor nucleus specific and not a result of changes in brainstem structure (Figures S3H–S3M).

We quantified the dispersal of motor nuclei using a nucleus coalescence index, comparing the experimental and control sides of the brainstem (Supplemental Experimental Procedures and Figures 4E and S3N–S3Q). This quantification revealed that all nuclei at r5 and r8 were significantly perturbed in their coalescence after NΔ390 expression. We next asked whether the observed desegregation of cranial motor nuclei after NΔ390 expression arose owing to a defect in radial migration of the neurons [22]. We characterized two migratory streams of motor neurons (see the Supplemental Experimental Procedures): one initially radial with motor neurons closely apposed to transitin radial glia, and a second lateral migration tangential to the orientation of transitin fibers. After NΔ390 expression, we did not observe differences in radial positioning of MNs in either migratory stream, indicating that NΔ390 perturbs motor nucleus coalescence and not radial migration of the neurons (Figures S3R–S3U).

### Differential Cadherin Expression Drives Cranial Motornucleogenesis

We next asked whether differential cadherin expression could drive specificity of motor nucleogenesis. At r5, the dorsal facial and the accessory abducens nuclei differ only in the expression of *cadherin-20* within the dorsal facial nucleus (Figure 2I). We hypothesized that expression of *cadherin-20* in the accessory abducens would alter its segregation from the facial nucleus. We misexpressed cadherin-20 by in ovo electroporation and followed its expression by a coelectroporated GFP reporter. Cadherin-20 overexpression had no effect on cranial motor neuron progenitor domains and motor neuron number, similar to that found after NΔ390 expression (Supplemental Experimental Procedures and Figures S4A–S4C). Overexpression of cadherin-20 resulted in mixing of facial and accessory abducens neurons at st30 compared to the control side of the brainstem (Figures 4F–4J), quantified using a neuronal mixing index (Supplemental Experimental Procedures). This suggests that equalization of cadherin expression profiles impairs segregation of nuclei. Cosegregation of accessory abducens and facial motor neurons was also observed when we expressed a cytoplasmically GFP-tagged cadherin-20 construct (Figures S4D–S4F).

We next expressed a dominant negative version of cadherin-20 [17, 27]. Again, we observed mixing of accessory abducens and facial nuclei at st30, suggestive of a normalization of cadherin function between both nuclei (Figures 4K–4O). The positioning of the abducens and facial nuclei and motor neuron number appeared normal after both cadherin-20 perturbations, indicating a specificity of action of cadherin-20 manipulations for the segregation of the facial and accessory abducens nuclei (Figures 4G–4I and 4L–4N, Supplemental Experimental Procedures, and data not shown). We next addressed the cell autonomy of each of these cadherin-20 perturbations using DNA constructs incorporating a nuclear β-galactosidase reporter. Mispositioned accessory abducens motor neurons were juxtaposed to facial motor neurons that expressed the dominant negative cadherin-20 (Figures S4G–S4K). Additionally, after cadherin-20 misexpression, mispositioned accessory abducens cells, but not normally positioned neurons, expressed the cadherin-20 construct (Figures S4L–S4P). Taken together, these data suggest a cell-autonomous role for cadherin-20 expression in the segregation of accessory abducens neurons from the facial motor nucleus.

To assess the specificity of cadherin expression to cranial motor nucleogenesis, we asked whether misexpression of cadherins shared by accessory abducens and facial motor neurons perturbed their segregation. Expression of N-cadherin, expressed by neither nucleus, or cadherin-6b, expressed by both nuclei, left nucleogenesis unperturbed (Figures 4P–4Y). These results argue for a specificity of cadherin function in the topographic organization of cranial motor nuclei. Thus, equalization of cadherin expression profiles results in comingling of nuclei, whereas general perturbation of cadherin function disrupts the formation of nuclei as coherent clusters of neurons. Despite differences in their progenitor domain origin, with different initial migratory paths and evolutionary origin of the structures that they innervate, both branchiomotor and somatomotor neurons employ differential cadherin function to drive topographic nucleogenesis.

Neuronal nuclei are an evolutionarily ancient mode of organization of neurons in the central nervous system, differing substantially from the organization of the lamina of the cortex [28, 29]. Our work suggests that cadherin function is a major contributor to driving nucleus segregation. Why do nuclei cluster in highly stereotyped positions in the CNS? Recent work has suggested that sensory afferent input to spinal motor neuron pools may require prior correct positioning of motor neurons within the ventral horn [30, 31]. Different cranial motor nuclei also occupy distinct positions and receive synaptic input from distinct sources. It seems likely that the stereotyped and highly reproducible positioning of these cranial motor nuclei may be required for appropriate afferent inputs. This precise topography therefore underlies the emergence of function of motor nuclei and motor circuits in the nervous system. Cadherin function is thus critical to the assembly of mature motor circuits. Cadherin expression is found throughout the developing nervous system, including in cortical areas of vertebrates and other neuronal nuclei [32, 33]. This expression also correlates with functional neuronal circuitry [34, 35]. Our results are suggestive of a general, evolutionarily conserved role for cadherin expression in the topographic ordering of neuronal nuclei and thus further suggest a broad role for cadherins in the assembly of functional neuronal circuits.

## Experimental Procedures

RNA in situ hybridization and in ovo electroporation followed standard procedures. Further details and details of identification of cranial motor nuclei, analysis of motor neuron migration, quantification of the results, and details of DNA constructs and antibodies used in the study are included as Supplemental Experimental Procedures.

## Author Contributions

All authors conceived the project, designed experiments, analyzed results, and wrote the manuscript. Experiments were performed by M.A., K.T., and W.M.

## Supplemental Information

Document S1. Supplemental Experimental Procedures and Figures S1–S4

Document S2. Article plus Supplemental Information
