# Supplementary material for: Central Topography of Cranial Motor Nuclei Controlled by Differential Cadherin Expression
Source: Curr Biol. 2014 Nov 3;24(21):2541–7. doi: 10.1016/j.cub.2014.08.067 (PMC4228048; doi:10.1016/j.cub.2014.08.067)
Supplement: Document S1. Supplemental Experimental Procedures and Figures S1–S4 [file mmc1.pdf]

Current Biology, Volume *24*

Supplemental Information

Central Topography of Cranial Motor Nuclei Controlled by Differential Cadherin Expression

Marc Astick, Kristina Tubby, Waleed M. Mubarak, Sarah C. Guthrie, and Stephen R. Price

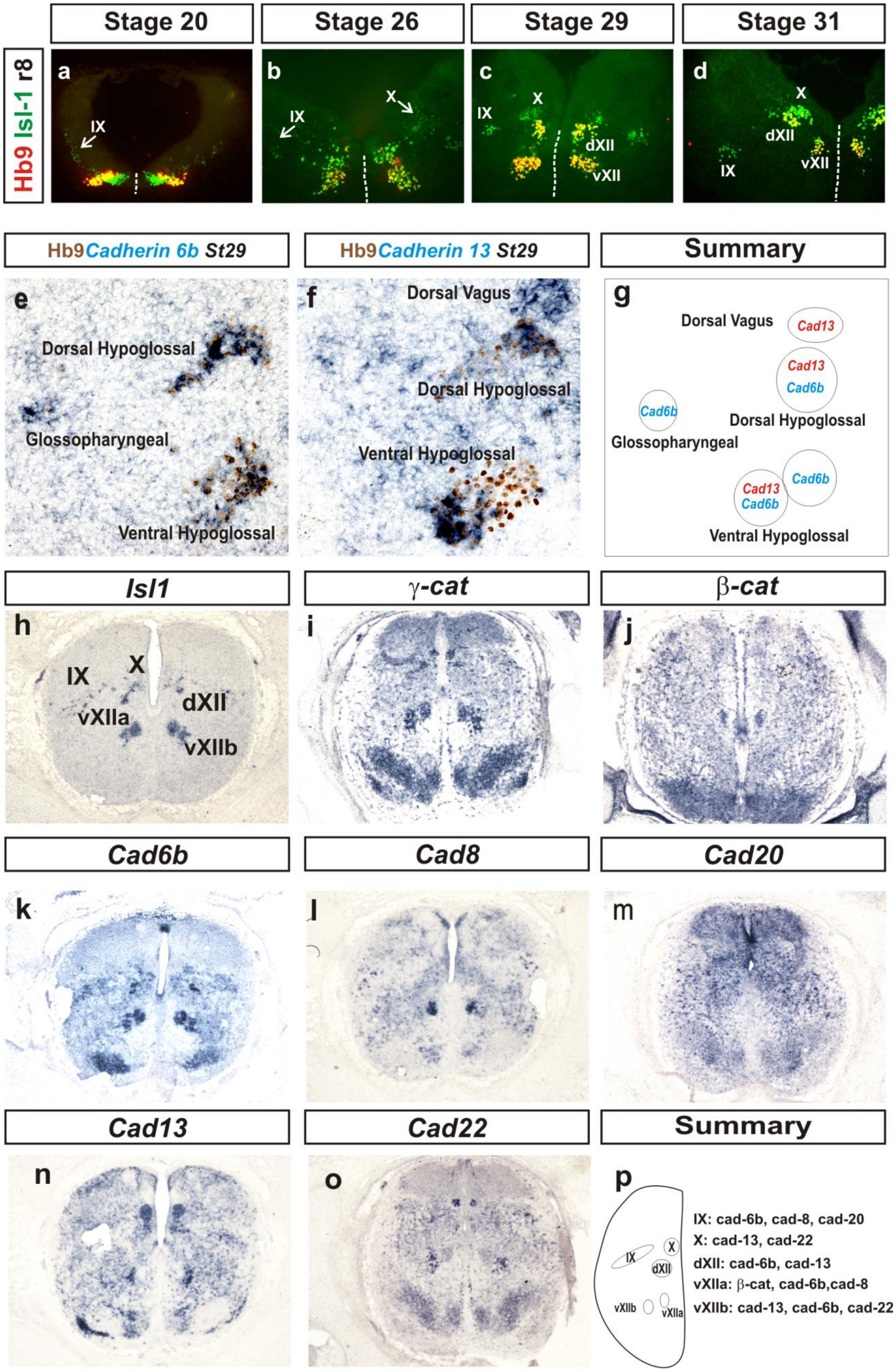

Stage 20
Stage 26
Stage 29
Stage 31
Hb9 Isl-1 r8
a
IX
b
IX
X
c
IX
X
dXII
vXII
d
X
dXII
IX
vXII
Hb9 Cadherin 6b St29
Hb9 Cadherin 13 St29
Summary
e
Dorsal Hypoglossal
Glossopharyngeal
Ventral Hypoglossal
f
Dorsal Vagus
Dorsal Hypoglossal
Ventral Hypoglossal
g
Dorsal Vagus
Cad13
Cad13
Cad6b
Cad6b
Dorsal Hypoglossal
Glossopharyngeal
Cad6b
Cad13
Cad6b
Ventral Hypoglossal
Isl1
γ-cat
β-cat
h
IX
X
dXII
vXIIa
vXIIb
i
j
Cad6b
Cad8
Cad20
k
l
m
Cad13
Cad22
Summary
n
o
p
IX
X
dXII
vXIIb
vXIIa
IX: cad-6b, cad-8, cad-20
X: cad-13, cad-22
dXII: cad-6b, cad-13
vXIIa: β-cat, cad-6b,cad-8
vXIIb: cad-13, cad-6b, cad-22

Figure S1

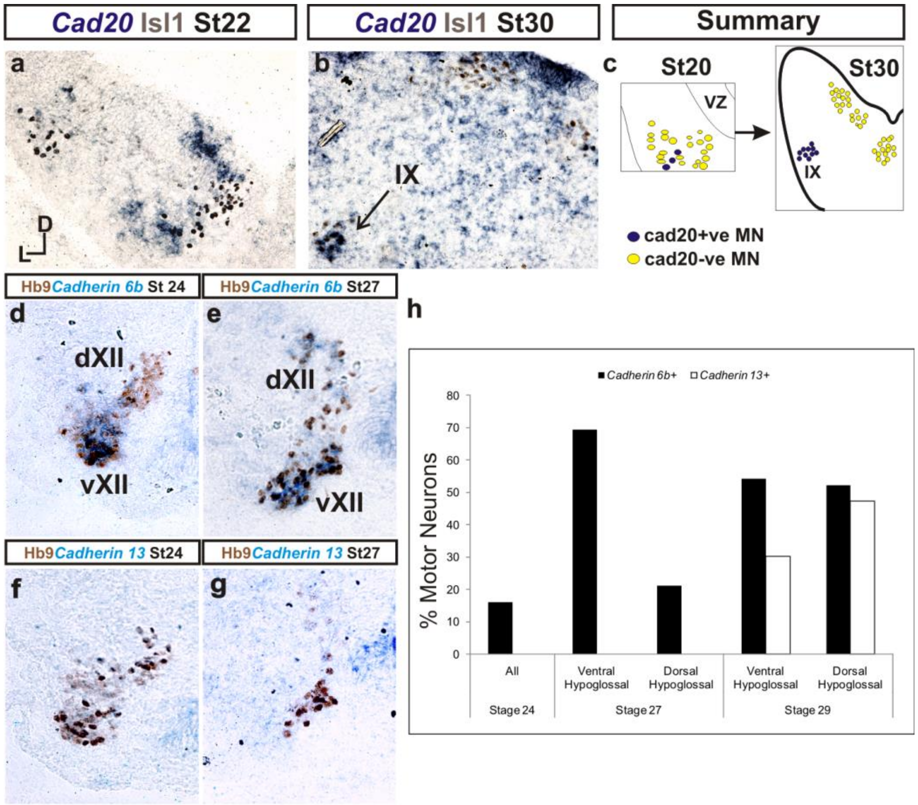
Cad20 Isl1 St22
Cad20 Isl1 St30
Summary
a
b
c
D
L
IX
St20
VZ
St30
IX
cad20+ve MN
cad20-ve MN
Hb9 Cadherin 6b St 24
Hb9 Cadherin 6b St27
d
e
dXII
vXII
dXII
vXII
Hb9 Cadherin 13 St24
Hb9 Cadherin 13 St27
f
g
h
Cadherin 6b+
Cadherin 13+
% Motor Neurons
80
70
60
50
40
30
20
10
0
All
Ventral Hypoglossal
Dorsal Hypoglossal
Ventral Hypoglossal
Dorsal Hypoglossal
Stage 24
Stage 27
Stage 29

Figure S2

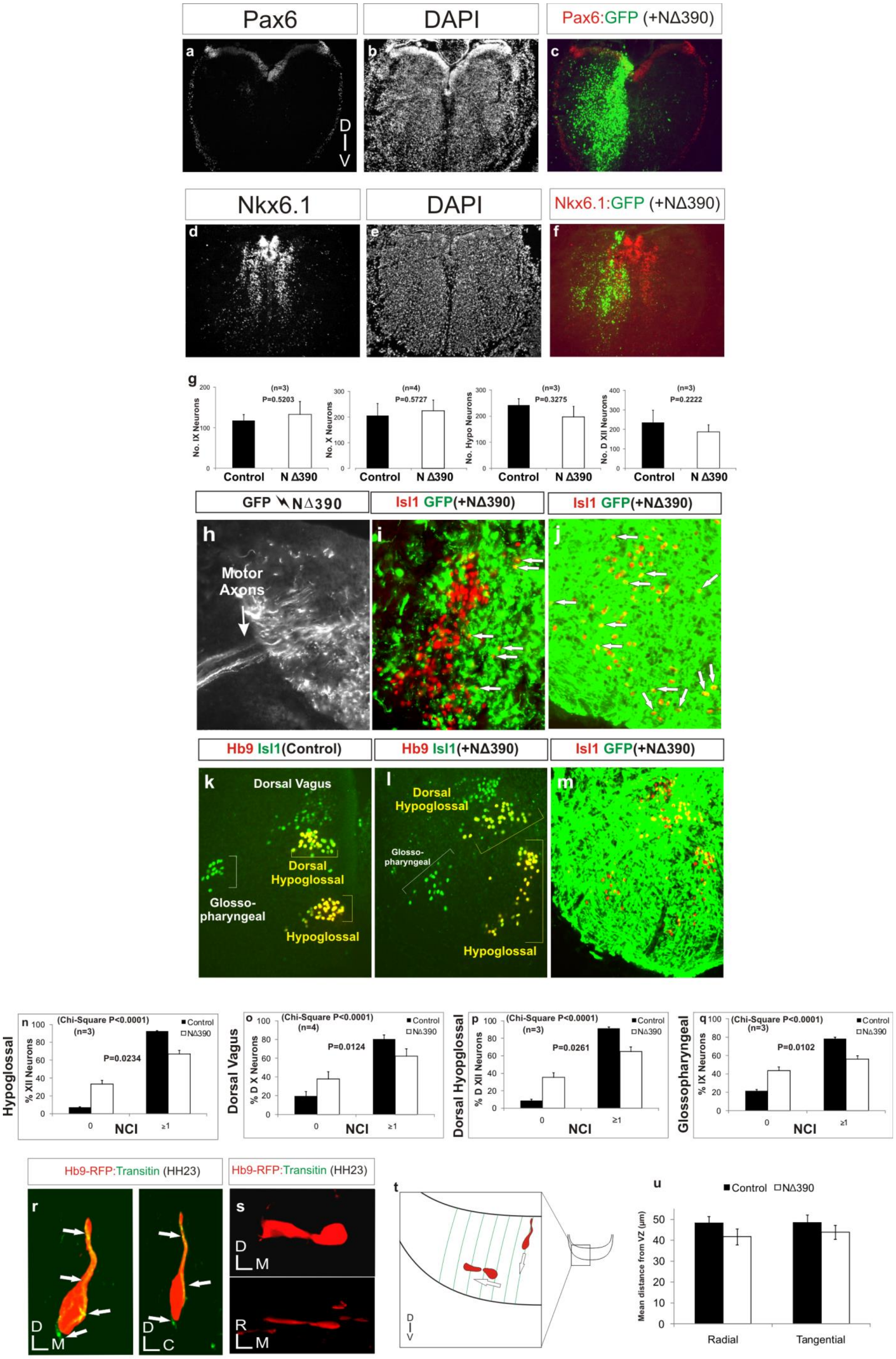
Pax6
DAPI
Pax6:GFP (+NΔ390)
a
b
c
D
V
Nkx6.1
DAPI
Nkx6.1:GFP (+NΔ390)
d
e
f
g
(n=3)
P=0.5203
No. IX Neurons
(n=4)
P=0.5727
No. X Neurons
(n=3)
P=0.3275
No. Hypo Neurons
(n=3)
P=0.2222
No. D XII Neurons
Control
N Δ390
GFP NΔ390
Isl1 GFP(+NΔ390)
Isl1 GFP(+NΔ390)
h
Motor Axons
i
j
Hb9 Isl1(Control)
Hb9 Isl1(+NΔ390)
Isl1 GFP(+NΔ390)
k
Dorsal Vagus
Dorsal Hypoglossal
Glosso-pharyngeal
Hypoglossal
l
Dorsal Hypoglossal
Glosso-pharyngeal
Hypoglossal
m
n
(Chi-Square P<0.0001)
(n=3)
Control
NΔ390
P=0.0234
Hypoglossal
% XII Neurons
NCI
o
(Chi-Square P<0.0001)
(n=4)
P=0.0124
Dorsal Vagus
% D X Neurons
p
(Chi-Square P<0.0001)
(n=3)
P=0.0261
Dorsal Hypoglossal
% D XII Neurons
q
(Chi-Square P<0.0001)
(n=3)
P=0.0102
Glossopharyngeal
% IX Neurons
Hb9-RFP:Transitin (HH23)
Hb9-RFP:Transitin (HH23)
r
D
M
D
C
s
D
M
R
M
t
D
V
u
Control NΔ390
Mean distance from VZ (µm)
Radial
Tangential

Figure S3

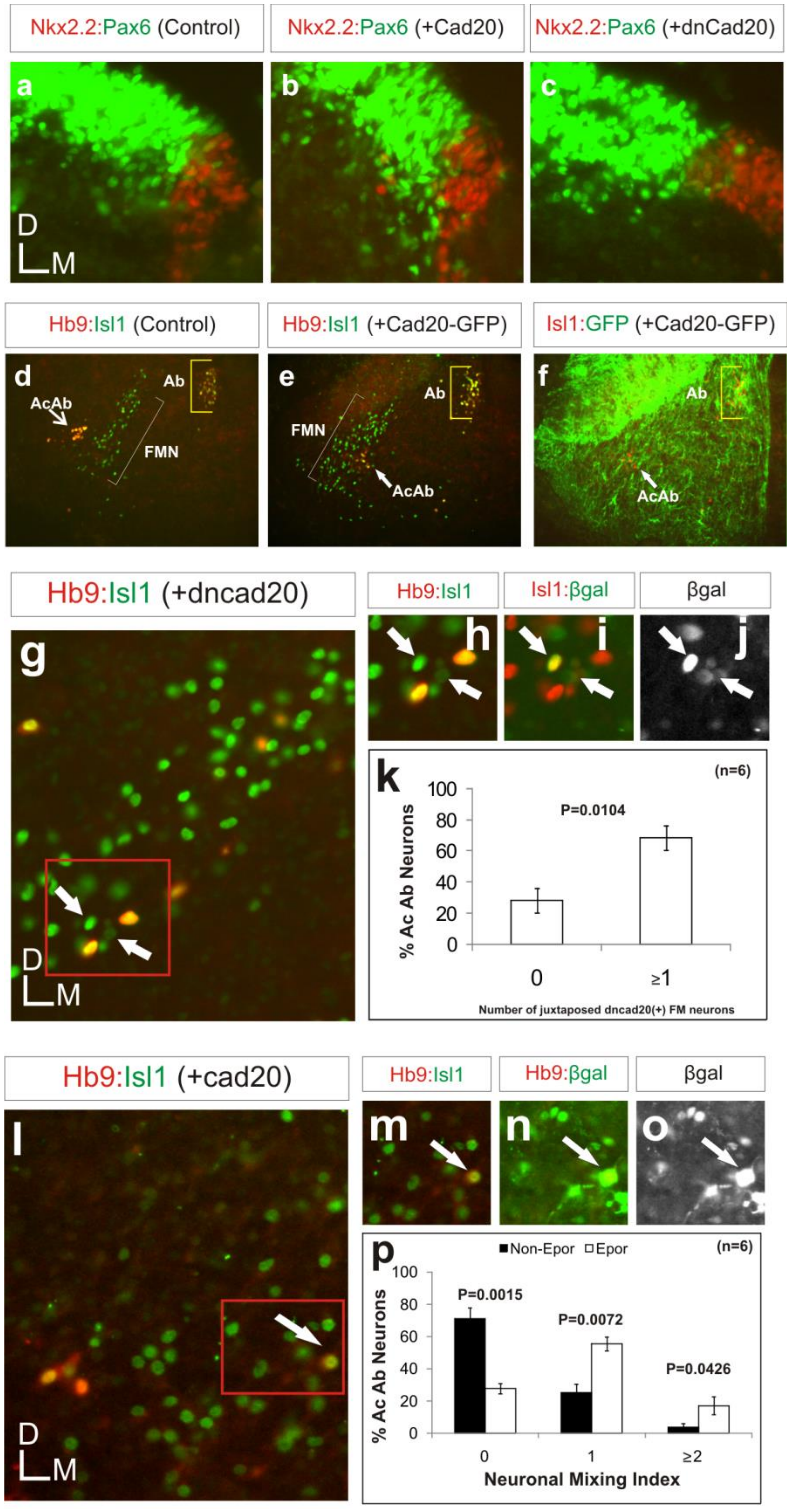
Nkx2.2:Pax6 (Control)
Nkx2.2:Pax6 (+Cad20)
Nkx2.2:Pax6 (+dnCad20)
a
b
c
D
M
Hb9:Isl1 (Control)
Hb9:Isl1 (+Cad20-GFP)
Isl1:GFP (+Cad20-GFP)
d
AcAb
Ab
FMN
e
Ab
FMN
AcAb
f
Ab
AcAb
Hb9:Isl1 (+dncad20)
g
D
M
Hb9:Isl1
Isl1:βgal
βgal
h
i
j
k
(n=6)
P=0.0104
% Ac Ab Neurons
100
80
60
40
20
0
0
≥1
Number of juxtaposed dncad20(+) FM neurons
Hb9:Isl1 (+cad20)
l
D
M
Hb9:Isl1
Hb9:βgal
βgal
m
n
o
p
Non-Epor
Epor
(n=6)
P=0.0015
P=0.0072
P=0.0426
% Ac Ab Neurons
100
80
60
40
20
0
0
1
≥2
Neuronal Mixing Index

Figure S4

Supplemental Figure Legends

Figure S1 related to figure 1. Nucleus segregation, cadherin and catenin expression at rhombomere 8.

Branchiomotor ($Hb9^{-}$/islet-1^{+}) and Somatomotor ($Hb9^{+}$islet-1^{+}) nuclei migrate, comingle and segregate between st20 (a), st26 (b), st29 (c) and st31 (d). This results in the hypoglossal (somatomotor) nucleus taking up a medial and also dorsal subgroup. The vagal nucleus (X) (visceromotor) is dorsal to the dorsal hypoglossal (dXII). The glossopharyngeal (IX) (brachio/visceromotor) nucleus is lateral to all those nuclei. White dotted lines show the midline. e. Costaining of Hb9 immunohistochemistry with *cadherin-6b in situ* hybridisation in r8 at stage 29 reveals that the ventral and dorsal hypoglossal and the glosopharyngeal nuclei all express cadherin-6b.

f. Costaining of Hb9 immunohistochemistry with *cadherin-13 in situ* hybridisation at stage 29 shows that the lateral part of the ventral hypoglossal, the dorsal hypoglossal and the vagal nuclei express cadherin-13.

g. Summary of *cadherin-6b* and *cadherin-13* expression at stage 29.

h. *Islet-1* in situ hybridization at st35 shows the ventral hypoglossal nucleus segregated into two distinct parts, the vagal nucleus and the glossopharyngeal nuclei. Glosopharyngeal nucleus spreads medio-laterally at this stage.

i-o *γ-catenin* (i) *β-catenin* (j), *cadherin-6b* (k), *cadherin-8* (l), *cadherin–20* (m) *cadherin-13* (n) and *cadherin-22* (0) expression by in situ hybridisation in st35 r8 cranial motor nuclei. Note that *γ-catenin* appears expressed in all cranial motor nuclei at r8 whereas *β-catenin* is expressed in only one part of the hypoglossal nucleus.

p. Summary of differential cadherin expression in r8 motor nuclei.

Figure S2 related to figure 2. Developmental profile of cadherins within cranial motor nuclei at r8.

a-c. *cadherin-20* expression in cranial motor neurons marked by islet-1 immunohistochemistry at r8 at st22 (a) and st30 (b). (c) summarises this data; at r8, *cadherin-20* appears in a small subset of motor neurons and this expression apparently persists throughout nucleus segregation. VZ is ventricular zone.

d,e. *cadherin-6b* in situ hybridisation with colabelling of Hb9 immunohistochemistry in r8 at stage 24 (d) and stage 27 (e).

f, g. *cadherin-13* in situ hybridisation with colabelling of Hb9 immunohistochemistry in r8 at stage 24 (f) and stage 27 (g). *Cadherin-13* is expressed in no motor neurons at r8 at either of these stages.

h. Summary of the expression of *cadherin-6b* and *cadherin-13* at r8 at stage 24 and within the dorsal and ventral hypoglossal nuclei at stages 27 and 29.

Figure S3 related to figure 3. Manipulation of cadherin function does not alter brainstem structure or motor neuron migration.

a-c Ventricular zone expression of Pax6 following NΔ390 expression in r5 at stage 29. a. Pax6 expression, b. DAPI staining of cell nuclei. c. Pax6 (red) and GFP (green) marking electroporation with the cadherin dominant negative construct.

d-f Expression of the Progenitor and postmitotic ventral interneuron marker Nkx6.1 following NΔ390 expression in r5 at stage 29. d. Nkx6.1 expression, e. DAPI staining of cell nuclei. f. Nkx6.1 (red) and GFP (Green) marking electroporation with the dominant negative construct.

g. Quantification of motor neuron number in r8 at stage 29 following NΔ390 expression compared to control.

h. GFP labeled motor axons following NΔ390 expression (indicated by arrow). The exit point and initial axonal trajectory is unaffected.

i, j. NΔ390 expression at r5 results in Facial Motor nucleus dissociation in a cell autonomous manner. $Islet^{+}$ /GFP^{+} motor neurons do not coalesce (indicated by arrows), $Islet^{+}$ /GFP^{-} motor neurons do coalesce (compare i with j)

k-m NΔ390-GFP expression disrupts nucleus clustering at r8 as assayed by Hb9 (k, l) and islet-1 (k-m) immunoreactivity. k. shows the control, l,m shows the results of NΔ390 expression marked by GFP immunofluorescence in m.

n-q Quantification of Nucleus Coalescence Index (NCI) of Hypoglossal (n), Vagal (o), Dorsal Hypoglossal (p) and Glosopharyngeal nuclei (q) comparing control to NΔ390 expression.

r-u. Migration of motor neurons is not altered by cadherin function disruption. Membrane RFP expressed under the control of the Hb9 promoter labels entire migrating somatic motor neurons. r. During radial migration MN's are closely associated with tranistin $^{+}$ radial glia (green). s. Motor neuron associations with radial glia are absent during tangential migration (two neurons are shown here). The morphology of radial vs tangentially migrating neurons is different (r, s).

t. Summary of migratory pathway of somatic motor neurons.

u. Quantification of the average position from the VZ of control RFP^{+} vs. $N\Delta390^{+}$ GFP^{+} RFP^{+} somatic motor neurons. Error bars show SEM.

Figure S4 related to figure 4. Cadherin 20-GFP, cadherin 20 and dominant negative cadherin 20 misexpression at r5.

a-c. Nkx2.2 and Pax6 label distinct motor neuron progenitor domains within the Ventricular zone. The structural integrity and gene expression is unaltered following cadherin manipulation; control (a), cadherin-20 misexpression (b), DN cadherin-20 expression(c).

d-f Clustering of Abducens and Facial motor nuclei are not altered following cadherin-20-GFP fusion expression. Hb9 (d, e), islet-1 (d-f) immunofluorescence in st30 control (d) or cadherin-20-GFP electroporated brainstem (e, f) marked by GFP immunofluorescence (green in f).

g-k. Cell autonomous analysis of AcAb/FMN mixing following dominant negative cadherin-20 expression. $Hb9^+$ /$islet^+$ AcAb neurons are mispositioned (g), magnification of boxed region (g), $islet^+\beta\text{-}Gal^+$ neurons confirm plasmid construct expression (h-j). Quantification shows that mispositioned AcAb neurons are in contact with ≥1 $islet^+\beta\text{-}Gal^+$ Facial Motor Neurons (k).

l-p. Cell autonomous analysis of AcAb/FMN mixing following cadherin 20 misexpression. $Hb9^+$ $islet^+$ AcAb neurons are mispositioned (l), magnification of boxed region (l), $Hb9^+\beta\text{-}Gal^+$ neurons confirm plasmid construct expression (m-o). Quantification using NMI showing that $Hb9^+\beta\text{-}Gal^+$ mispositioned AcAb neurons exhibit increased aberrant mixing behavior when compared with control ($Hb9^+\beta\text{-}Gal^-$) neurons (p). Error bars show SEM.

Supplemental Experimental Procedures

Chick Embryo Preparation

Fertilised Brown Bovan Gold Hen's eggs (Henry Stewart Farms, UK) were incubated in a forced draft incubator at 38°C and staged (as in [14]). All embryos were treated in accordance with the Animals (Scientific Procedures) Act of 1986, UK.

In Situ Hybridisation Histochemistry.

Digoxigenin (DIG)-labelled anti-sense cRNA probes were used for in situ hybridisation histochemistry on 15 μm thick cryostat sections as in [17]. Dual in situ hybridisation with immunohistochemistry was performed by replacing the proteinase K treatment with a 30 minute incubation of the slide sections with 0.1% Triton X-100 in phosphate buffered saline (PBS) (pH7.4). Development of the RNA in situ was performed as normal followed by incubation with the primary antibody for either islet-1 or Hb9 for 16 hours at 4°C. Following this, secondary HRP conjugated goat anti mouse antibodies were applied and ImPact DAB substrate reagent (Vector labs) was used to reveal HRP immunohistochemistry following manufacturers instructions.

Identification of cranial nuclei.

The entire hindbrain was analysed in transverse section with both Hb9 and islet-1 immunofluorescence. Maps of positioning of somatomotor and branchiomotor neurons in the rostro-caudal extent of the brainstem [5] allowed the assignment of rough identity of the cranial motor nuclei that contribute axons to the VIth, VIIth, IXth, X^{th} and XIIth cranial nerves. Further assignment of nucleus identity was based on maps of transverse sections of the adult chicken

brainstem [10]. Cadherin expression within each nucleus was confirmed for a selected series of cadherins through double immunohistochemistry and in situ hybridization for Hb9 or islet-1 with the cadherin antisense cRNA probe.

Generation of Expression Constructs

Full-length cDNAs for chick cadherin-20, N-cadherin, cadherin-6b, Dominant negative cadherin-20, GFP or NΔ390 were cloned into a pCAGGS or pCAGGS ires nuclear localized β-galactosidase vector. Cadherin-20-GFP was generated by removing the stop codon and cloning the cDNA for eGFP immediately downstream. Dominant negative cadherin-20 (DNcad-20) was generated by removing the cDNA of cadherin-20 from aa 684 to aa 798, which retains part of the juxtamembrane domain. Other constructs used in this work include transposase integrated doxycycline inducible N-cadherinΔ390 [S1-S3] and Hb9:RFP [S4] which was generated and kindly gifted by I. Lieberam.

In Ovo Electroporation

Expression of cDNAs was achieved by *in ovo* electroporation using an ECM830 electro-squareporator (BTX Inc.). ~0.1μl of DNA construct (1-10μg/μl in H_2O with 0.1% Fast Green (Sigma)) was pressure injected into the lumen of the brain stem. Five 30 Volt electrical pulses of 50ms duration evenly spaced over a 5 second period were applied by placing electrodes adjacent to each side of the head of the embryo. Embryos were electroporated at HH stages 12-18 and analyzed at HH stages 25-32. For NΔ390 experiments, doxycycline hyclate (200μl) at a concentration of 0.25 μg/ml in H_20 was applied from stage 20 onwards.

Immunohistochemistry.

Antibodies used in this study were: Rabbit (R) anti-GFP (Invitrogen, 1/1000), R anti-RFP (1/1000), R anti Hb9 (1/5000), Guinea pig (GP) anti-islet1(2) (1/20000), Mouse (M) anti GFP (1/100), R anti β-galactosidase (Abcam, 1/1000) or chicken anti β-galactosidase (abcam, 1/1000), Alkaline phosphatase conjugated Sheep anti-DIG Fab fragments (Roche) (1/5000). The following mouse monoclonal antibodies were purchased from the Developmental Studies Hybridoma Bank, 2D6 and 4D5 (anti islet-1), 5C10 (anti MNR2/Hb9), F55A10 (anti Nkx 6.1), Pax6 (anti Pax6), 74.5A5 (anti Nkx 2.2), EAP3 (anti transitin). Immunocytochemistry was performed essentially as described in [17]. Cryostat sections mounted on superfrost plus glass slides were incubated in PBS for 5 minutes followed by incubation in block solution (PBS with 1% Goat serum (Sigma) and 0.1% Triton-X-100) for 30 minutes at 20°C. This solution was replaced by antibody diluted in block solution and incubated for 12 to 16 hours at 4°C. Following three washes of 5 minutes in PBS, fluorescent conjugated secondary antibodies were incubated with the sections for 30 minutes at 20°C in block solution, washed and mounted with vectashield fluorescent mounting medium (Vector Labs).

Analysis of Radial Migration of Cranial Motor Neurons.

We electroporated a DNA construct that drives Red Fluorescent Protein (RFP) under the control of the murine HB9 promoter [S4]. All RFP positive motor neurons (>100) expressed HB9. We characterized two migratory streams of motor neurons, those closely apposed to transitin expressing radial glia and a second stream that migrated tangentially to the transitin fibres. The morphology of the neurons in each stream differed; those radially migrating

possessing an extended trailing process. We assessed radial distance from the ventricle of the soma of each HB9:RFP expressing motor neuron dependent on whether it was electroporated by the NΔ390 construct or not and which migratory stream the motor neuron was found to be in. The mean distances from the ventricular zone of all four bins of motor neurons were not significantly different from each other. This suggests that the radial migration of motor neurons is not affected by NΔ390 expression for either those neurons migrating along transitin radial glia or those migrating tangentially to the radial glia.

Image Acquisition

Images were acquired on a Nikon Eclipse E80i fluorescence microscope equipped with a Nikon DS5M and Hamammatsu ORCA ER digital camera or on a Leica SPE confocal microscope.

Quantification.

Cell counts were performed across the entire rostrocaudal extent of the nuclei as identified by positive Hb9 and islet 1 staining. Sections were 15μm thick and were cut in series of four; as such each cell count represents approximately ¼ of the total cell number of each nucleus. Cell numbers from the sections of each embryo were collated and each embryo counts as one point in the data set. Mean values were calculated from the total cell number counted for each embryo and standard error was calculated across this data set (n=5 embryos, except where stated). A paired Student's t-Test was performed comparing total cell number counted within each embryo between internal control and experimental hemispheres (contralateral to electroporated side, n=5, except where stated). NΔ390 expression did not alter the total number

of cranial motor neurons at rhombomere 5 (168±58 versus 188±100 (control vs NΔ390 somatic motor neurons), p=0.19 student's t-test; 614±57 versus 584±33 (control vs NΔ390 total motor neurons, p=0.414 student's t-test; n=3 embryos). Additionally, the total number of accessory abducens neurons was unaffected by both cadherin-20 manipulations (42±7 vs 41±4 (control vs cad-20 electroporation), p=0.885 student's t-test; 48±4 vs 44±4 (control vs dominant negative cad-20 electroporation), p=0.60 student's t-test). Similarly, manipulations of cad-6b and N-cad maintained normal neuron number (60±15 versus 51±14 accessory abducens neurons cad6b electroporation, p=0.70 and 46±2 vs 48±13 for N-cad electroporation)

The Neuronal Mixing index (NMI) was calculated essentially as described [17]. In brief, each single neuron of either the accessory abducens or the facial nucleus was scored by the number of neurons of the other nucleus that surrounded it and allocated to an appropriate bin. The percentage of neurons of each type in each bin is reported. Statistical analysis was performed using a student's t-test between experimental and control bins in turn and through a χ^2 analysis of the distribution of neurons across all the bins. For analysis of cell autonomy of cadherin-20 manipulations, each mispositioned neuron was divided dependent upon the presence or absence of GFP/β-galactosidase. NMI was then performed following Cadherin 20 misexpression and a direct comparison made between electroporated and non-electroporated neurons. Following dominant negative cadherin 20 expression, mispositioned neurons were placed in bins based upon their juxtaposition to Islet-1^+ Facial Motor neurons; either 0 (GFP/β-gal $^-$/ Islet-1 $^+$) or ≥1 (GFP/β-gal $^+$/ Islet-1 $^+$) . Comparison of the two bins indicated that a mispositioned neuron was likely to be juxtaposed to a facial motor neuron electroporated with the cadherin-20 dominant negative construct.

For the Nucleus Coalescence Index (NCI), individual neurons of the same type were chosen and the number of neurons surrounding it that were of the same identity counted. Neurons in the zero bin are thus separated from their neighbours. Neurons in the remaining bin had at least one neighbour adjacent and were deemed to be within the nucleus proper.

Supplemental References

S1. Kawakami, K., and Noda, T. (2004) Transposition of the Tol2 element, an Ac-like element from the Japanese medaka fish Oryzias latipes, in mouse embryonic stem cells. Genetics *166***,** 895-899.

S2. Sato, Y., *et al*, (2007) Stable integration and conditional expression of electroporated transgenes in chicken embryos. Dev Biol *305***,** 616-624.

S3. Watanabe, T., *et al*, (2007) Tet-on inducible system combined with in ovo electroporation dissects multiple roles of genes in somitogenesis of chicken embryos. Dev Biol. *305***,** 625-636

S4. Wichterle H., Lieberam I., Porter J. A., and Jessell T. M. (2002) Directed differentiation of embryonic stem cells into motor neurons. Cell *110*, 385–397.
