# Supplementary material for: Central Topography of Cranial Motor Nuclei Controlled by Differential Cadherin Expression
Source: Curr Biol. 2014 Nov 3;24(21):2541–7. doi: 10.1016/j.cub.2014.08.067 (PMC4228048; doi:10.1016/j.cub.2014.08.067)
Supplement: Document S2. Article plus Supplemental Information [file mmc2.pdf]

Current Biology 24, 2541–2547, November 3, 2014 ©2014 The Authors http://dx.doi.org/10.1016/j.cub.2014.08.067

Report

Central Topography of Cranial Motor Nuclei Controlled by Differential Cadherin Expression

Marc Astick,[1] Kristina Tubby,[1] Waleed M. Mubarak,[1] Sarah Guthrie,[2] and Stephen R. Price[1,*]
[1]Research Department of Cell and Developmental Biology, University College London, Gower Street, London WC1E 6BT, UK
[2]MRC Centre for Developmental Neurobiology, 4th Floor New Hunt's House, Kings College, Guy's Campus, London SE1 1UL, UK

Summary

Neuronal nuclei are prominent, evolutionarily conserved features of vertebrate central nervous system (CNS) organization [1]. Nuclei are clusters of soma of functionally related neurons and are located in highly stereotyped positions. Establishment of this CNS topography is critical to neural circuit assembly. However, little is known of either the cellular or molecular mechanisms that drive nucleus formation during development, a process termed nucleogenesis [2–5]. Brainstem motor neurons, which contribute axons to distinct cranial nerves and whose functions are essential to vertebrate survival, are organized exclusively as nuclei. Cranial motor nuclei are composed of two main classes, termed branchiomotor/visceromotor and somatomotor [6]. Each of these classes innervates evolutionarily distinct structures, for example, the branchial arches and eyes, respectively. Additionally, each class is generated by distinct progenitor cell populations and is defined by differential transcription factor expression [7, 8]; for example, Hb9 distinguishes somatomotor from branchiomotor neurons. We characterized the time course of cranial motornucleogenesis, finding that despite differences in cellular origin, segregation of branchiomotor and somatomotor nuclei occurs actively, passing through a phase of each being intermingled. We also found that differential expression of cadherin cell adhesion family members uniquely defines each motor nucleus. We show that cadherin expression is critical to nucleogenesis as its perturbation degrades nucleus topography predictably.

Results and Discussion

To investigate the mechanisms of somatomotor versus branchiomotor nucleogenesis, we focused our attention on rhombomere 5 (r5) and r8 of the brainstem of the chicken embryo. Cranial motor neurons at these levels are born within these rhombomeres, and there is little rostrocaudal migration of the motor neurons while they take up their stereotyped positions. In all, eight distinct motor nuclei are generated at r5 and r8, and these motor neurons contribute axons to five cranial nerves. Four distinct motor nuclei, two somatic and two branchiomotor, are located in r5. There, the somatic abducens and accessory abducens nuclei project axons via the VIth cranial nerve to the lateral rectus muscle and retractor bulbi muscles of the eye [9, 10], respectively. The branchiomotor dorsal and ventral divisions of the facial motor nucleus [11] contribute axons to the VIIth cranial nerve and control beak opening (dorsal nucleus) or innervate suprahyoid muscles of the tongue (ventral nucleus) [6, 12]. At r8, the glossopharyngeal (IXth) and vagal (Xth) (both branchio-/visceromotor) and hypoglossal (XIIth) (somatomotor) cranial nerves receive axons from four spatially segregated clusters of motor neurons.

Cranial Motor Nucleogenesis Requires an Active Segregation of the Motor Nuclei

We first characterized the time course of cranial motor nucleogenesis at r5 by analyzing immunofluorescence for Hb9^{+} and Islet-1^{+} somatomotor neurons (SMNs) and Hb9^{-}, Islet-1^{+} branchiomotor neurons (BMNs) (Figure 1). We found that the early trajectories of the migration of facial and accessory abducens neurons were different with adjacent paths radially followed by distinct paths dorsally (Figure 1A) [13]. However, at stage 26 (st26) [14], the migratory streams of both the presumptive facial and accessory abducens nuclei have converged and the neurons of each nucleus are intermingled (Figure 1B). By st31, the accessory abducens has segregated from the facial nucleus, residing dorsolaterally to it, and the loose aggregate of the facial nucleus has separated into dorsal and ventral subdivisions (Figures 1C–1E). In contrast, the abducens neurons migrated only radially and formed a loose aggregate of cells close to the midline, which became better defined between st26 and st31 (Figure 1).

A similar scheme of motor nucleogenesis was observed at r8 (Figure S1 available online). SMNs and BMNs are generated in adjacent progenitor domains (Figure S1A). However, at st26, considerable mixing of the somatomotor XIIth neurons with the branchiomotor/visceromotor neurons is observed (Figure S1B). The four distinct groupings of IXth, Xth and dorsal and ventral divisions of the XIIth nuclei at r8 emerge by st29 to st32 (Figures S1C and S1D).

Thus, at both r5 and r8, characteristic migratory streams of motor neurons resulted in initially scattered and intermingled cell groups that segregated in a highly stereotyped manner. Despite distinctions in birthplace and initial paths of migration, branchiomotor and somatomotor neurons pass through a phase of being mixed and then sort out from one another. Additionally, both somatic and branchiomotor nuclei initially form loosely defined nuclei and coalesce into characteristic, distinct locations within the brainstem. Thus, cranial motor nucleogenesis involves an active process of segregation of the nuclei, despite early differences in progenitor cell location. We next asked how the specificity by which cranial motor nuclei sort from one another could be driven molecularly.

Differential Cadherin Expression Defines Cranial Motor Nuclei

We focused our attention on the cadherin family of cell adhesion molecules as candidates to drive the sorting and segregation of cranial motor nuclei, as they play key roles in the

*Correspondence: stephen.price@ucl.ac.uk
This is an open access article under the CC BY license (http://creativecommons.org/licenses/by/3.0/).
CrossMark

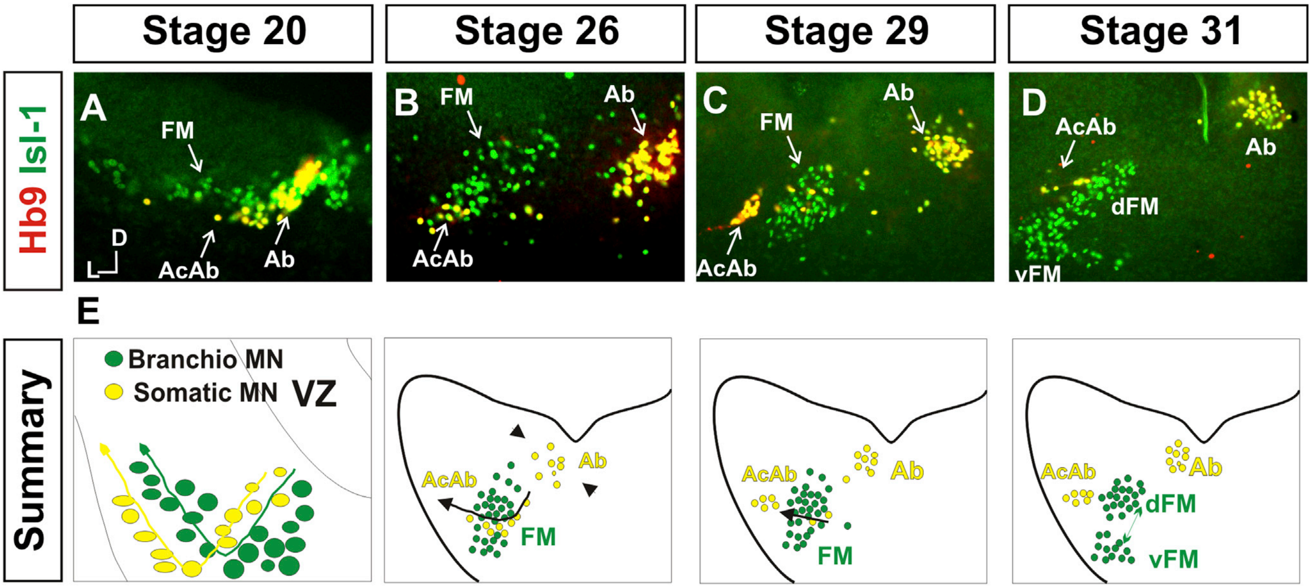

Figure 1. Development of Motor Nucleus Formation at Rhombomere 5

(A–D) Branchiomotor (Hb9$^-$/Islet-1$^+$) and Somatomotor (Hb9$^+$Islet-1$^+$) neurons in r5 at st20 (A), st26 (B), st29 (C), and st31 (D). Arrows show accessory abducens (AcAb), abducens (Ab) or facial motorneurons. Abducens neurons form initially a relatively undefined cluster (A and B) that becomes more coherent by st31 (D). Accessory abducens cells and facial neurons have distinct migration paths which converge and then segregate and cluster by st31. (E) Summary. VZ is ventricular zone, FM is facial motor neurons, and d and v are dorsal and ventral, respectively.
See also Figure S1.

organization of spinal lateral motor column neurons [15–21]. We surveyed classical cadherin expression at st35 and found differential expression of six cadherins (*cadherin-6b* [22], *cadherin-8*, *cadherin-11*, *cadherin-13*, *cadherin-20*, and *cadherin-22*) in the cranial motor nuclei of r5 and r8 (Figures 2A–2I and S1E–S1P). For example, at r5, all four nuclei expressed *cadherin-11*, whereas three nuclei expressed *cadherin-6b* and two nuclei expressed *cadherin-8* and/or *cadherin-13*. *cadherin-22* was expressed in only the ventral facial nucleus, and *cadherin-20* was expressed in only the dorsal facial nucleus. Overall, each nucleus was defined by a unique combination of cadherins (Figure 2H). At r8, each of the four distinct groupings of motor neurons also expressed different combinations of cadherins (Figure S1; summarized in Figure S1P). Notably, based on dual immunohistochemistry for Hb9 expression with cadherin in situ hybridization, we were able to distinguish two subsets of the ventral hypoglossal motor nucleus on the basis of the differential expression of *cadherin-13* and *cadherin-6b*, with *cadherin-13* being predominantly expressed in a lateral grouping of the ventral hypoglossal from st29 (Figures S1E–S1G). These two subsets of the ventral hypoglossal become more separated by st36 (Figure S1H), indicating that cadherin expression can delineate nuclei prior to their becoming physically distinguishable clusters of cells. Based on these data, no two cranial motor nuclei at either r5 or r8 shared the same cadherin expression profile (Figures 2H and S1P).

Importantly, γ-*catenin*, an armadillo family member required for cadherin function, was expressed in all motor nuclei at r5 and r8 (Figures 2I and S1I). However, we only observed expression of the related β-*catenin* within the medial subset of the ventral hypoglossal nucleus at r8. This suggests that the major cytoplasmic partner of classical cadherins in cranial motor neurons is γ-catenin and not β-catenin as is commonly assumed.

Cadherin Expression in Cranial Motor Nuclei Is Highly Dynamic during Development

We next asked whether the developmental profile of cadherin expression was consistent with a role in cranial motor nucleogenesis. We assayed cadherin expression at r5 and r8 by double in situ hybridization with islet-1 or Hb9 immunohistochemistry between stages 20 and 30. (Figures 3 and S2). At r5, *cadherin-20* expression was initiated in the majority of motor neurons soon after they were born and was refined from st25 to st30, being downregulated in all neurons other than the dorsal facial nucleus, resulting in the mature pattern of expression (Figures 3A–3D; quantified in Figure 3E and summarized in Figure 3F). In contrast, at r8, *cadherin-20* expression was initiated in a restricted subset of motor neurons, this being maintained through the period of nucleogenesis (Figures S2A–S2C). *cadherin-6b* was expressed in the presumptive ventral hypoglossal at st24 but only became expressed in the dorsal hypoglossal by st27. In contrast, *cadherin-13* expression was not apparent in the hypoglossal nuclei until st29 (Figures S2D–S2H and S1F; summarized in Figures S1G and S2H). Thus, cadherin expression is highly dynamic within cranial motor neurons, and its refinement to a mature pattern coincides with the period of nucleogenesis. Cadherins are thus good candidates to drive cranial motor nucleogenesis.

Cadherin Function Drives Nucleus Coalescence

To test whether cadherin expression controls cranial motor nucleogenesis, we perturbed general cadherin function through the expression of the dominant negative isoform NΔ390 (Figures 4A–4E) Expression was confirmed by GFP immunofluorescence driven by the electroporated plasmid [23–26] (Figure 4D). After NΔ390 expression, the progenitor domains from which motor neurons arise appeared to be unaffected at either r5 or r8 (Figures S3A–S3F). Consistent with this, the total number of motor neurons was not affected

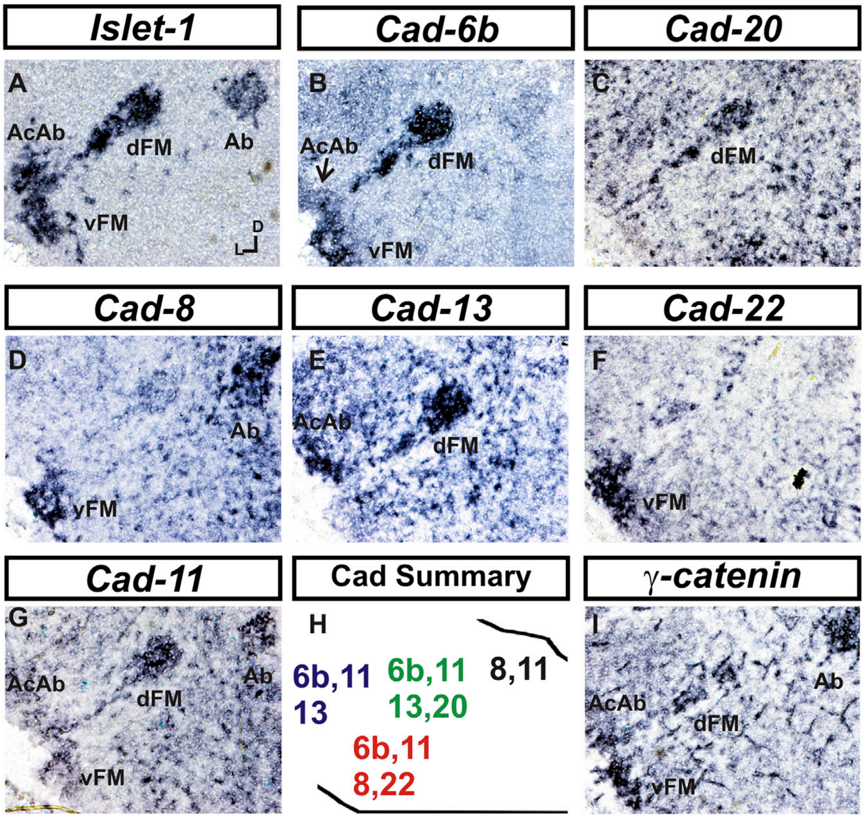

Figure 2. Cadherin and Catenin Expression in r5 at stage 35

(A) *Islet-1* in situ hybridization shows the topography of nucleus positioning of abducens (Ab), accessory abducens (AcAb), dorsal facial nucleus (dFM), and ventral facial motor nucleus (vFM).

(B–H) *cad-6b* (B), *cad–20* (C), *cad-8* (D), *cad-13* (E), *cad–22* (F), and *cad-11* (G) expression on adjacent sections. A summary of differential cadherin expression in r5 motor nuclei is shown in (H). Note that no two nuclei share the same cadherin combination and that the accessory abducens and dorsal facial nuclei differ by the expression of *cad-20* in the dorsal facial nucleus.

(I) γ-*catenin* expression in all four motor nuclei at r5.

See also Figure S2.

(Supplemental Experimental Procedures and Figure S3G), and motor axons projected out of the brainstem normally (Figure S3H and data not shown). This suggests that cadherin perturbation does not affect motor neuron differentiation and allowed us to assess the effect of NΔ390 expression on motor nucleus formation. We observed a failure of all motor nuclei at r5 and r8 to coalesce after NΔ390 expression (Figures 4A–4E and S3I–S3Q). For example, the accessory abducens and facial motor nuclei at r5 were scattered over a larger area after NΔ390 expression compared to the control (see the yellow brackets in Figures 4B and 4C). This effect was not observed where motor neurons were nonelectroporated (Figures S3I and S3J), suggesting that cell-autonomous cadherin function is essential for nucleogenesis. Taken together, these results suggest that the observed phenotype is motor nucleus specific and not a result of changes in brainstem structure (Figures S3H–S3M).

We quantified the dispersal of motor nuclei using a nucleus coalescence index, comparing the experimental and control sides of the brainstem (Supplemental Experimental Procedures and Figures 4E and S3N–S3Q). This quantification revealed that all nuclei at r5 and r8 were significantly perturbed in their coalescence after NΔ390 expression. We next asked whether the observed desegregation of cranial motor nuclei after NΔ390 expression arose owing to a defect in radial migration of the neurons [22]. We characterized two migratory streams of motor neurons (see the Supplemental Experimental Procedures): one initially radial with motor neurons closely apposed to transitin radial glia, and a second lateral migration tangential to the orientation of transitin fibers. After NΔ390 expression, we did not observe differences in radial positioning of MNs in either migratory stream, indicating that NΔ390 perturbs motor nucleus coalescence and not radial migration of the neurons (Figures S3R–S3U).

Differential Cadherin Expression Drives Cranial Motornucleogenesis

We next asked whether differential cadherin expression could drive specificity of motor nucleogenesis. At r5, the dorsal facial and the accessory abducens nuclei differ only in the expression of *cadherin-20* within the dorsal facial nucleus (Figure 2I). We hypothesized that expression of *cadherin-20* in the accessory abducens would alter its segregation from the facial nucleus. We misexpressed cadherin-20 by in ovo electroporation and followed its expression by a coelectroporated GFP reporter. Cadherin-20 overexpression had no effect on cranial motor neuron progenitor domains and motor neuron number, similar to that found after NΔ390 expression (Supplemental Experimental Procedures and Figures S4A–S4C). Overexpression of cadherin-20 resulted in mixing of facial and accessory abducens neurons at st30 compared to the control side of the brainstem (Figures 4F–4J), quantified using a neuronal mixing index (Supplemental Experimental Procedures). This suggests that equalization of cadherin expression profiles impairs segregation of nuclei. Cosegregation of accessory abducens and facial motor neurons was also observed when we expressed a cytoplasmically GFP-tagged cadherin-20 construct (Figures S4D–S4F).

We next expressed a dominant negative version of cadherin-20 [17, 27]. Again, we observed mixing of accessory abducens and facial nuclei at st30, suggestive of a normalization of cadherin function between both nuclei (Figures 4K–4O). The positioning of the abducens and facial nuclei and motor neuron number appeared normal after both cadherin-20 perturbations, indicating a specificity of action of cadherin-20 manipulations for the segregation of the facial and accessory abducens nuclei (Figures 4G–4I and 4L–4N, Supplemental Experimental Procedures, and data not shown). We next addressed the cell autonomy of each of these cadherin-20 perturbations using DNA constructs incorporating a nuclear β-galactosidase reporter. Mispositioned accessory abducens motor neurons were juxtaposed to facial motor neurons that expressed the dominant negative cadherin-20

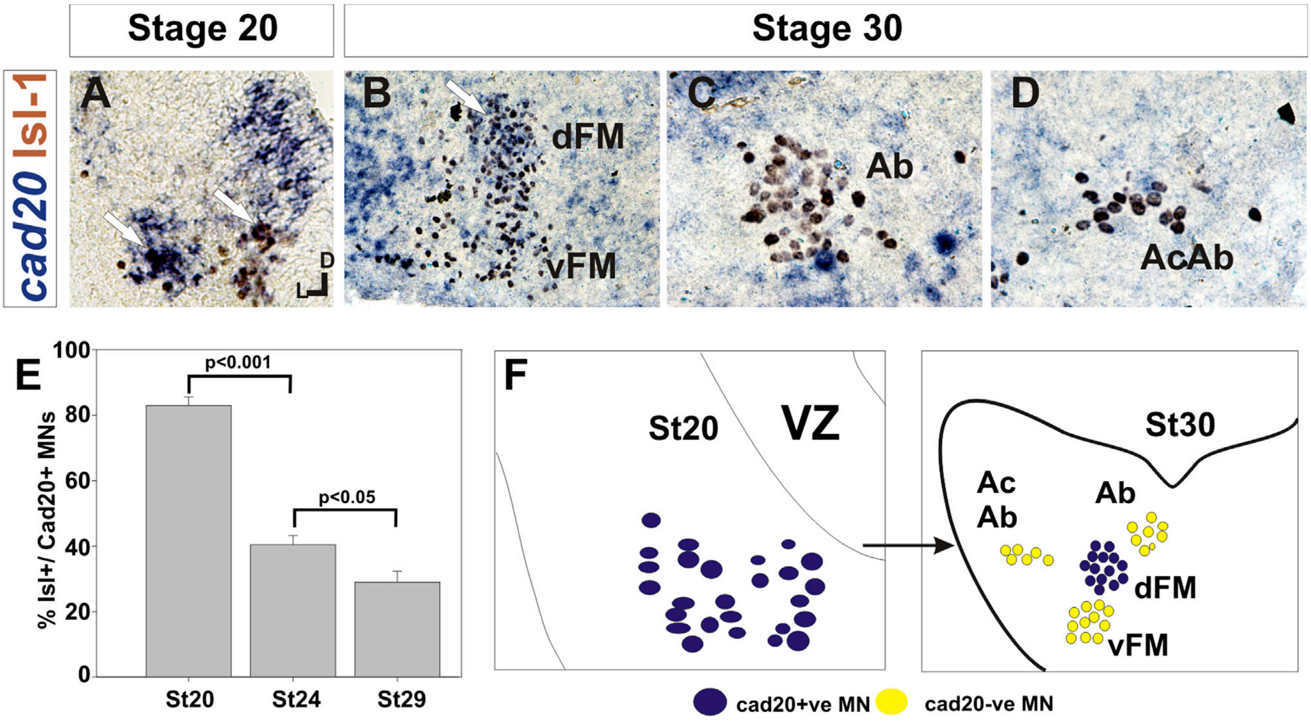

Figure 3. Developmental Time Course of *cadherin-20* Expression at r5

(A–D) *cadherin-20* in situ hybridization with islet-1 immunohistochemistry expression at st20 (A) and st30 (B–D). For clarity, higher-magnification images of facial (B), abducens (C), and accessory abducens (D) nuclei are shown. Arrows indicate Cadherin 20⁺ islet-1⁺ neurons as examples.

(E) Quantification of the percentage of islet-1 motor neurons at r5 that express *cadherin-20* at st20, st24, and st29. n = 4 embryos at each stage. Student's t test p values shown above the bar graphs. Error bars indicate the SEM.

(F) Summary of this expression. *cadherin-20* is expressed in the majority of motor neurons at r5 at st20, and this expression is refined to the mature pattern by st30. VZ, ventricular zone.

See also Figure S3.

(Figures S4G–S4K). Additionally, after cadherin-20 misexpression, mispositioned accessory abducens cells, but not normally positioned neurons, expressed the cadherin-20 construct (Figures S4L–S4P). Taken together, these data suggest a cell-autonomous role for cadherin-20 expression in the segregation of accessory abducens neurons from the facial motor nucleus.

To assess the specificity of cadherin expression to cranial motor nucleogenesis, we asked whether misexpression of cadherins shared by accessory abducens and facial motor neurons perturbed their segregation. Expression of N-cadherin, expressed by neither nucleus, or cadherin-6b, expressed by both nuclei, left nucleogenesis unperturbed (Figures 4P–4Y). These results argue for a specificity of cadherin function in the topographic organization of cranial motor nuclei. Thus, equalization of cadherin expression profiles results in comingling of nuclei, whereas general perturbation of cadherin function disrupts the formation of nuclei as coherent clusters of neurons. Despite differences in their progenitor domain origin, with different initial migratory paths and evolutionary origin of the structures that they innervate, both branchiomotor and somatomotor neurons employ differential cadherin function to drive topographic nucleogenesis.

Neuronal nuclei are an evolutionarily ancient mode of organization of neurons in the central nervous system, differing substantially from the organization of the lamina of the cortex [28, 29]. Our work suggests that cadherin function is a major contributor to driving nucleus segregation. Why do nuclei cluster in highly stereotyped positions in the CNS? Recent work has suggested that sensory afferent input to spinal motor neuron pools may require prior correct positioning of motor neurons within the ventral horn [30, 31]. Different cranial motor nuclei also occupy distinct positions and receive synaptic input from distinct sources. It seems likely that the stereotyped and highly reproducible positioning of these cranial motor nuclei may be required for appropriate afferent inputs. This precise topography therefore underlies the emergence of function of motor nuclei and motor circuits in the nervous system. Cadherin function is thus critical to the assembly of mature motor circuits. Cadherin expression is found throughout the developing nervous system, including in cortical areas of vertebrates and other neuronal nuclei [32, 33]. This expression also correlates with functional neuronal circuitry [34, 35]. Our results are suggestive of a general, evolutionarily conserved role for cadherin expression in the topographic ordering of neuronal nuclei and thus further suggest a broad role for cadherins in the assembly of functional neuronal circuits.

Experimental Procedures

RNA in situ hybridization and in ovo electroporation followed standard procedures. Further details and details of identification of cranial motor nuclei, analysis of motor neuron migration, quantification of the results, and details of DNA constructs and antibodies used in the study are included as Supplemental Experimental Procedures.

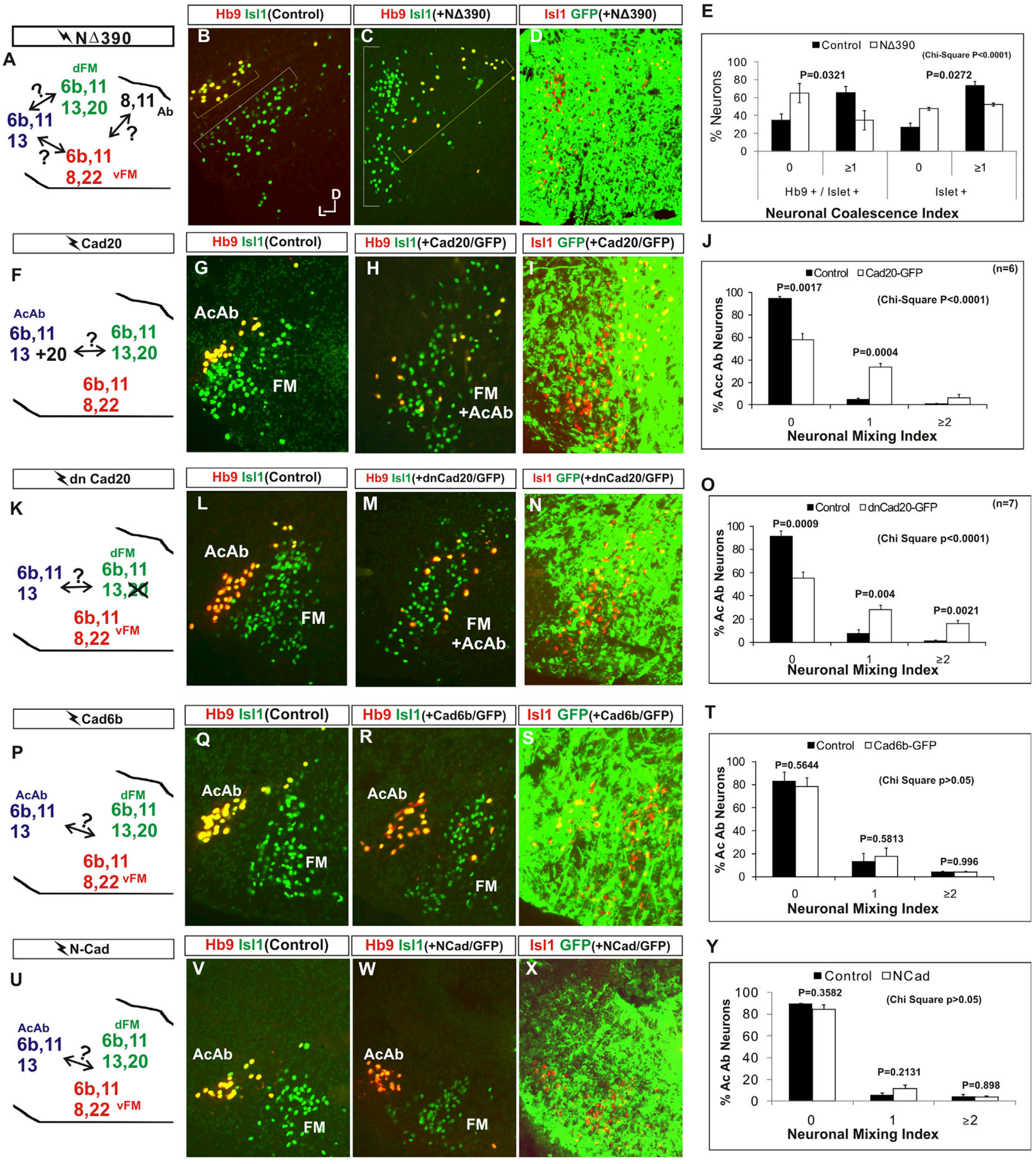

Figure 4. Manipulation of Cadherin Gene Function or Expression Perturbs Cranial Motor Nucleus Topography at r5

(A–E) NΔ390-GFP expression disrupts nucleus clustering at r5 as assayed by Hb9 (B and C) and islet-1 (B and D) immunoreactivity. (A) shows a schematic of the experiment, (B) shows the internal control side of the brainstem, (C) and (D) show the results of NΔ390 expression, and (E) shows quantification of nucleus coalescence using a nucleus coalescence index (see the Supplemental Experimental Procedures). The brackets in (B) and (C) show the spatial extent of facial (FM) and abducens (Ab) nuclei in the control and experimental sides.

(F–J) cad-20/GFP coexpression results in the mixing of accessory abducens (AcAb) and facial motor (FM) nuclei assayed by Hb9 (G and H) and islet-1 (G–I) immunoreactivity. (F) shows a schematic of the experiment, (G) shows internal control, and (H) and (I) show results of cad-20 expression, marked by GFP in (I). Quantification of the nucleus mixing (see Supplemental Experimental Procedures) is shown in (J). p values for a Student's t test of each bin are shown above the graph bars. The chi-squared p value for the entire distribution is $p < 0.0001$, with two degrees of freedom.

(K–O) DNcad-20/GFP coexpression results in a similar mixing of nuclei to cad-20 expression. (K) shows a schematic of the experiment, (L) shows the internal control, and (M) and (N) show the effect of DNcad-20 expression assessed by Hb9 (L and M) and islet-1 (L and N) expression. Electroporation is marked by

(legend continued on next page)

Supplemental Information

Supplemental Information includes Supplemental Experimental Procedures and four figures and can be found with this article online at http://dx.doi.org/10.1016/j.cub.2014.08.067.

Author Contributions

All authors conceived the project, designed experiments, analyzed results, and wrote the manuscript. Experiments were performed by M.A., K.T., and W.M.

Acknowledgments

We thank P. Salinas, S. Wilson, and J. Parnavelas for critical comments on the manuscript and S. Boast for many helpful discussions. The hybridomas obtained from the Developmental Studies Hybridoma Bank were developed under the auspices of the NICHD and maintained by the Department of Biology, University of Iowa. M.A. was supported by an MRC PhD studentship and K.T. by a Wellcome Trust PhD studentship. This work was supported by a grant from the Wellcome Trust (094399/B/10/Z) to S.G. and S.R.P.

Received: November 20, 2012
Revised: August 4, 2014
Accepted: August 29, 2014
Published: October 9, 2014

References

1. Cajal, S.R.Y. (1995). Histology of the Nervous System of Man and Vertebrates, *Volume 1* (New York: Oxford University Press), p. 14.
2. Agarwala, S., and Ragsdale, C.W. (2002). A role for midbrain arcs in nucleogenesis. Development *129*, 5779–5788.
3. Kawauchi, D., Taniguchi, H., Watanabe, H., Saito, T., and Murakami, F. (2006). Direct visualization of nucleogenesis by precerebellar neurons: involvement of ventricle-directed, radial fibre-associated migration. Development *133*, 1113–1123.
4. Hasan, K.B., Agarwala, S., and Ragsdale, C.W. (2010). PHOX2A regulation of oculomotor complex nucleogenesis. Development *137*, 1205–1213.
5. Nishida, K., Nakayama, K., Yoshimura, S., and Murakami, F. (2011). Role of Neph2 in pontine nuclei formation in the developing hindbrain. Mol. Cell. Neurosci. *46*, 662–670.
6. Guthrie, S. (2007). Patterning and axon guidance of cranial motor neurons. Nat. Rev. Neurosci. *8*, 859–871.
7. Varela-Echavarría, A., Tucker, A., Püschel, A.W., and Guthrie, S. (1997). Motor axon subpopulations respond differentially to the chemorepellents netrin-1 and semaphorin D. Neuron *18*, 193–207.
8. Pattyn, A., Vallstedt, A., Dias, J.M., Sander, M., and Ericson, J. (2003). Complementary roles for Nkx6 and Nkx2 class proteins in the establishment of motoneuron identity in the hindbrain. Development *130*, 4149–4159.
9. Stibbe, E.P. (1928). A Comparative Study of the Nictitating Membrane of Birds and Mammals. J. Anat. *62*, 159–176.
10. Labandeira-Garcia, J.L., Guerra-Seijas, M.J., Segade, L.A., and Suarez-Nuñez, J.M. (1987). Identification of abducens motoneurons, accessory abducens motoneurons, and abducens internuclear neurons in the chick by retrograde transport of horseradish peroxidase. J. Comp. Neurol. *259*, 140–149.
11. Jungherr, E.L., Helmboldt, C.F., and Timmins, P. (1969). The Neuroanatomy of the Domestic Foul (Gallus domesticus) (Amherst: University of Massachusetts).
12. Wild, J.M., and Zeigler, H.P. (1980). Central representation and somatotopic organization of the jaw muscles within the facial and trigeminal nuclei of the pigeon (Columba livia). J. Comp. Neurol. *192*, 175–201.
13. Ju, M.J., Aroca, P., Luo, J., Puelles, L., and Redies, C. (2004). Molecular profiling indicates avian branchiomotor nuclei invade the hindbrain alar plate. Neuroscience *128*, 785–796.
14. Hamburger, V., and Hamilton, H.L. (1992). A series of normal stages in the development of the chick embryo. 1951. Dev. Dyn. *195*, 231–272.
15. Bello, S.M., Millo, H., Rajebhosale, M., and Price, S.R. (2012). Catenin-dependent cadherin function drives divisional segregation of spinal motor neurons. J. Neurosci. *32*, 490–505.
16. Demireva, E.Y., Shapiro, L.S., Jessell, T.M., and Zampieri, N. (2011). Motor neuron position and topographic order imposed by β- and γ-catenin activities. Cell *147*, 641–652.
17. Price, S.R., De Marco Garcia, N.V., Ranscht, B., and Jessell, T.M. (2002). Regulation of motor neuron pool sorting by differential expression of type II cadherins. Cell *109*, 205–216.
18. Patel, S.D., Ciatto, C., Chen, C.P., Bahna, F., Rajebhosale, M., Arkus, N., Schieren, I., Jessell, T.M., Honig, B., Price, S.R., and Shapiro, L. (2006). Type II cadherin ectodomain structures: implications for classical cadherin specificity. Cell *124*, 1255–1268.
19. Nollet, F., Kools, P., and van Roy, F. (2000). Phylogenetic analysis of the cadherin superfamily allows identification of six major subfamilies besides several solitary members. J. Mol. Biol. *299*, 551–572.
20. Hirano, S., Suzuki, S.T., and Redies, C. (2003). The cadherin superfamily in neural development: diversity, function and interaction with other molecules. Front. Biosci. *8*, d306–d355.
21. Takeichi, M. (2007). The cadherin superfamily in neuronal connections and interactions. Nat. Rev. Neurosci. *8*, 11–20.
22. Barnes, S.H., Price, S.R., Wentzel, C., and Guthrie, S.C. (2010). Cadherin-7 and cadherin-6B differentially regulate the growth, branching and guidance of cranial motor axons. Development *137*, 805–814.
23. Kintner, C. (1992). Regulation of embryonic cell adhesion by the cadherin cytoplasmic domain. Cell *69*, 225–236.
24. Fujimori, T., and Takeichi, M. (1993). Disruption of epithelial cell-cell adhesion by exogenous expression of a mutated nonfunctional N-cadherin. Mol. Biol. Cell *4*, 37–47.
25. Levine, E., Lee, C.H., Kintner, C., and Gumbiner, B.M. (1994). Selective disruption of E-cadherin function in early Xenopus embryos by a dominant negative mutant. Development *120*, 901–909.
26. Tanabe, K., Takahashi, Y., Sato, Y., Kawakami, K., Takeichi, M., and Nakagawa, S. (2006). Cadherin is required for dendritic morphogenesis and synaptic terminal organization of retinal horizontal cells. Development *133*, 4085–4096.
27. Ozawa, M., and Kemler, R. (1998). The membrane-proximal region of the E-cadherin cytoplasmic domain prevents dimerization and negatively regulates adhesion activity. J. Cell Biol. *142*, 1605–1613.
28. Rakic, P. (2006). A century of progress in corticoneurogenesis: from silver impregnation to genetic engineering. Cereb. Cortex *16* (*Suppl 1*), i3–i17.
29. Lui, J.H., Hansen, D.V., and Kriegstein, A.R. (2011). Development and evolution of the human neocortex. Cell *146*, 18–36.
30. Sürmeli, G., Akay, T., Ippolito, G.C., Tucker, P.W., and Jessell, T.M. (2011). Patterns of spinal sensory-motor connectivity prescribed by a dorsoventral positional template. Cell *147*, 653–665.
31. Jessell, T.M., Sürmeli, G., and Kelly, J.S. (2011). Motor neurons and the sense of place. Neuron *72*, 419–424.
32. Krishna-K, Nuernberger, M., Weth, F., and Redies, C. (2009). Layer-specific expression of multiple cadherins in the developing visual cortex (V1) of the ferret. Cereb. Cortex *19*, 388–401.

GFP immunofluorescence in (N). Quantification of nucleus mixing is shown in (O). p values for a Student's t test of each bin is shown above the graph bars. The chi-squared p value for the entire distribution is $p < 0.0001$, with two degrees of freedom.

(P–T) Cadherin-6b (whose expression is found in both accessory abducens and facial motor nuclei) has no effect on AcAb and FMN segregation at st30 when misexpressed. (P) shows a summary of experiment, (Q) shows the control side of the brainstem, and (R) and (S) show the experimental side of the brainstem. Electroporation is marked by GFP in (S). Quantification of neuronal mixing index is shown in (T).

(U–Y) N-cadherin (whose expression is found in neither accessory abducens and facial motor nuclei) has no effect on AcAb and FMN segregation at st30 when misexpressed. (U) shows a summary of experiment, (V) shows the control side of the brainstem, and (W) and (X) show the experimental side of the brainstem. Electroporation is marked by GFP in (X). Quantification of neuronal mixing index is shown in (Y).

Error bars indicate the SEM. See also Figure S4.

33. Becker, T., and Redies, C. (2003). Internal structure of the nucleus rotundus revealed by mapping cadherin expression in the embryonic chicken visual system. J. Comp. Neurol. *467*, 536–548.
34. Suzuki, S.C., Inoue, T., Kimura, Y., Tanaka, T., and Takeichi, M. (1997). Neuronal circuits are subdivided by differential expression of type-II classic cadherins in postnatal mouse brains. Mol. Cell. Neurosci. *9*, 433–447.
35. Neudert, F., and Redies, C. (2008). Neural circuits revealed by axon tracing and mapping cadherin expression in the embryonic chicken cerebellum. J. Comp. Neurol. *509*, 283–301.

Current Biology, Volume *24*

Supplemental Information

Central Topography of Cranial Motor Nuclei Controlled by Differential Cadherin Expression

Marc Astick, Kristina Tubby, Waleed M. Mubarak, Sarah C. Guthrie, and Stephen R. Price

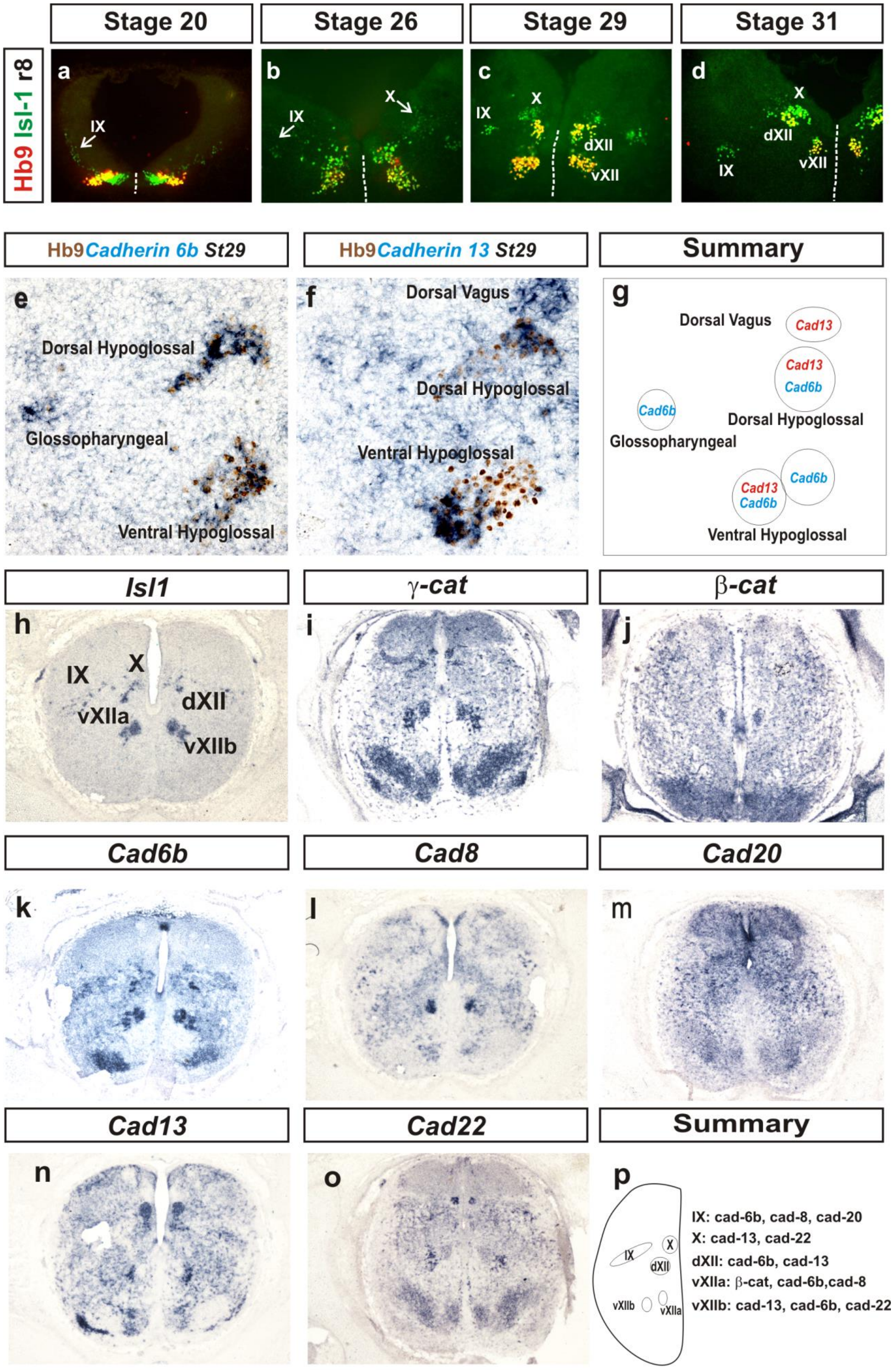
Stage 20
Stage 26
Stage 29
Stage 31
Hb9 Isl-1 r8
a
IX
b
IX
X
c
IX
X
dXII
vXII
d
X
dXII
vXII
IX
Hb9Cadherin 6b St29
Hb9Cadherin 13 St29
Summary
e
Dorsal Hypoglossal
Glossopharyngeal
Ventral Hypoglossal
f
Dorsal Vagus
Dorsal Hypoglossal
Ventral Hypoglossal
g
Dorsal Vagus
Cad13
Cad13
Cad6b
Cad6b
Dorsal Hypoglossal
Glossopharyngeal
Cad6b
Cad13
Cad6b
Ventral Hypoglossal
Isl1
γ-cat
β-cat
h
IX
X
dXII
vXIIa
vXIIb
i
j
Cad6b
Cad8
Cad20
k
l
m
Cad13
Cad22
Summary
n
o
p
IX
X
dXII
vXIIb
vXIIa
IX: cad-6b, cad-8, cad-20
X: cad-13, cad-22
dXII: cad-6b, cad-13
vXIIa: β-cat, cad-6b,cad-8
vXIIb: cad-13, cad-6b, cad-22

Figure S1

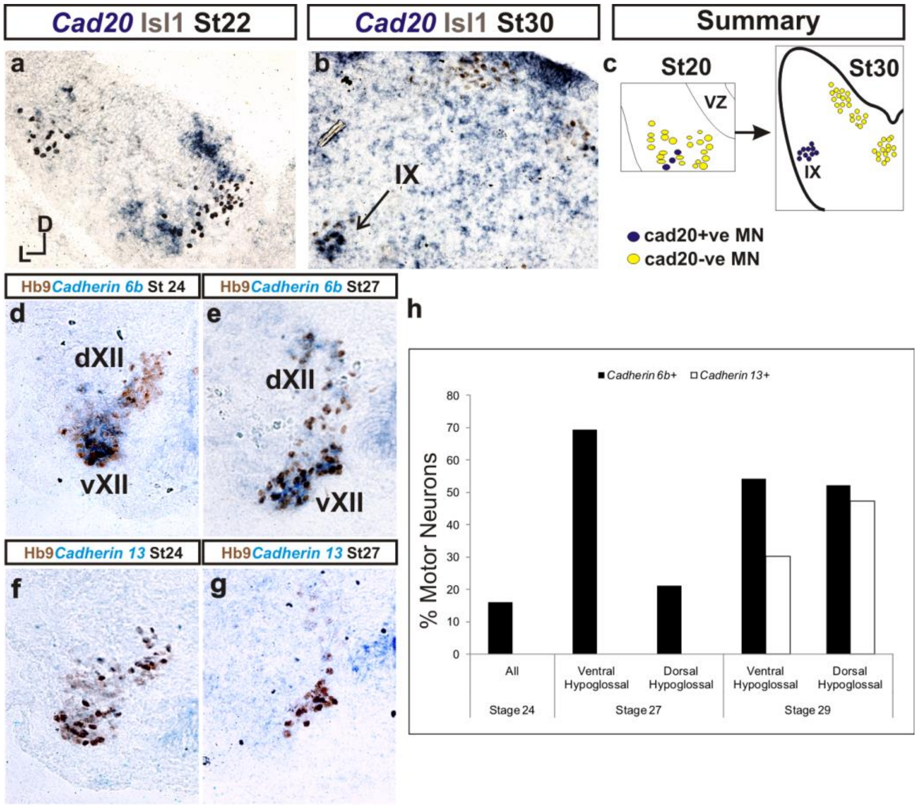
Cad20 Isl1 St22
Cad20 Isl1 St30
Summary
a
b
c
D
L
IX
St20
VZ
St30
IX
cad20+ve MN
cad20-ve MN
Hb9 Cadherin 6b St 24
Hb9 Cadherin 6b St27
d
e
h
dXII
vXII
dXII
vXII
Hb9 Cadherin 13 St24
Hb9 Cadherin 13 St27
f
g
Cadherin 6b+
Cadherin 13+
% Motor Neurons
80
70
60
50
40
30
20
10
0
All
Ventral Hypoglossal
Dorsal Hypoglossal
Ventral Hypoglossal
Dorsal Hypoglossal
Stage 24
Stage 27
Stage 29

Figure S2

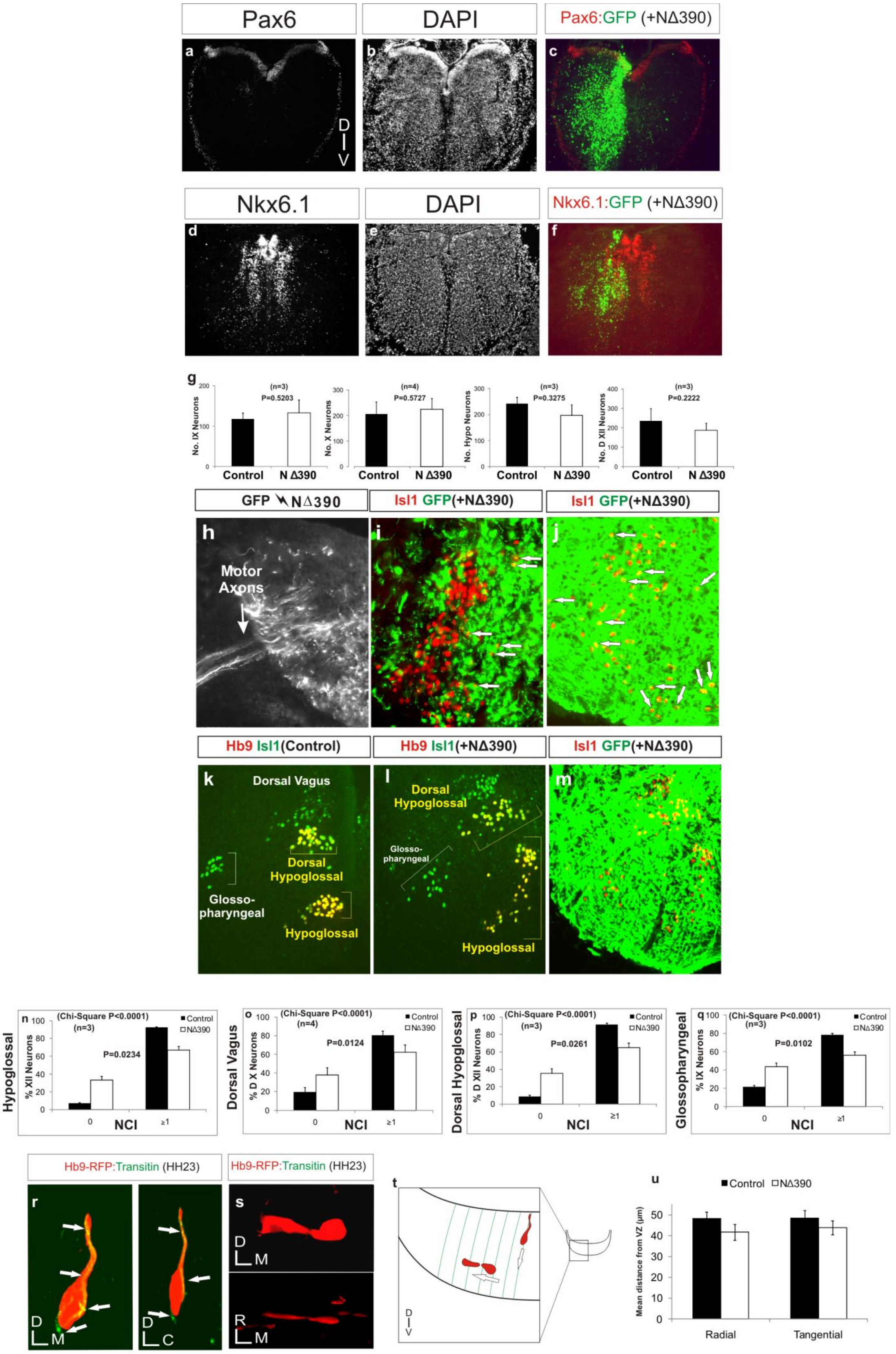
Pax6
DAPI
Pax6:GFP (+NΔ390)
a
b
c
D
V
Nkx6.1
DAPI
Nkx6.1:GFP (+NΔ390)
d
e
f
g
(n=3)
P=0.5203
No. IX Neurons
(n=4)
P=0.5727
No. X Neurons
(n=3)
P=0.3275
No. Hypo Neurons
(n=3)
P=0.2222
No. D XII Neurons
Control
N Δ390
GFP NΔ390
Isl1 GFP(+NΔ390)
Isl1 GFP(+NΔ390)
h
Motor
Axons
i
j
Hb9 Isl1(Control)
Hb9 Isl1(+NΔ390)
Isl1 GFP(+NΔ390)
k
Dorsal Vagus
Dorsal
Hypoglossal
Glosso-
pharyngeal
Hypoglossal
l
Dorsal
Hypoglossal
Glosso-
pharyngeal
Hypoglossal
m
n
Hypoglossal
(Chi-Square P<0.0001)
(n=3)
P=0.0234
% XII Neurons
o
Dorsal Vagus
(Chi-Square P<0.0001)
(n=4)
P=0.0124
% D X Neurons
p
Dorsal Hypoglossal
(Chi-Square P<0.0001)
(n=3)
P=0.0261
% D XII Neurons
q
Glossopharyngeal
(Chi-Square P<0.0001)
(n=3)
P=0.0102
% IX Neurons
Control
NΔ390
NCI
0
≥1
Hb9-RFP:Transitin (HH23)
Hb9-RFP:Transitin (HH23)
r
D
M
D
C
s
D
M
R
M
t
D
V
u
Control
NΔ390
Mean distance from VZ (µm)
Radial
Tangential

Figure S3

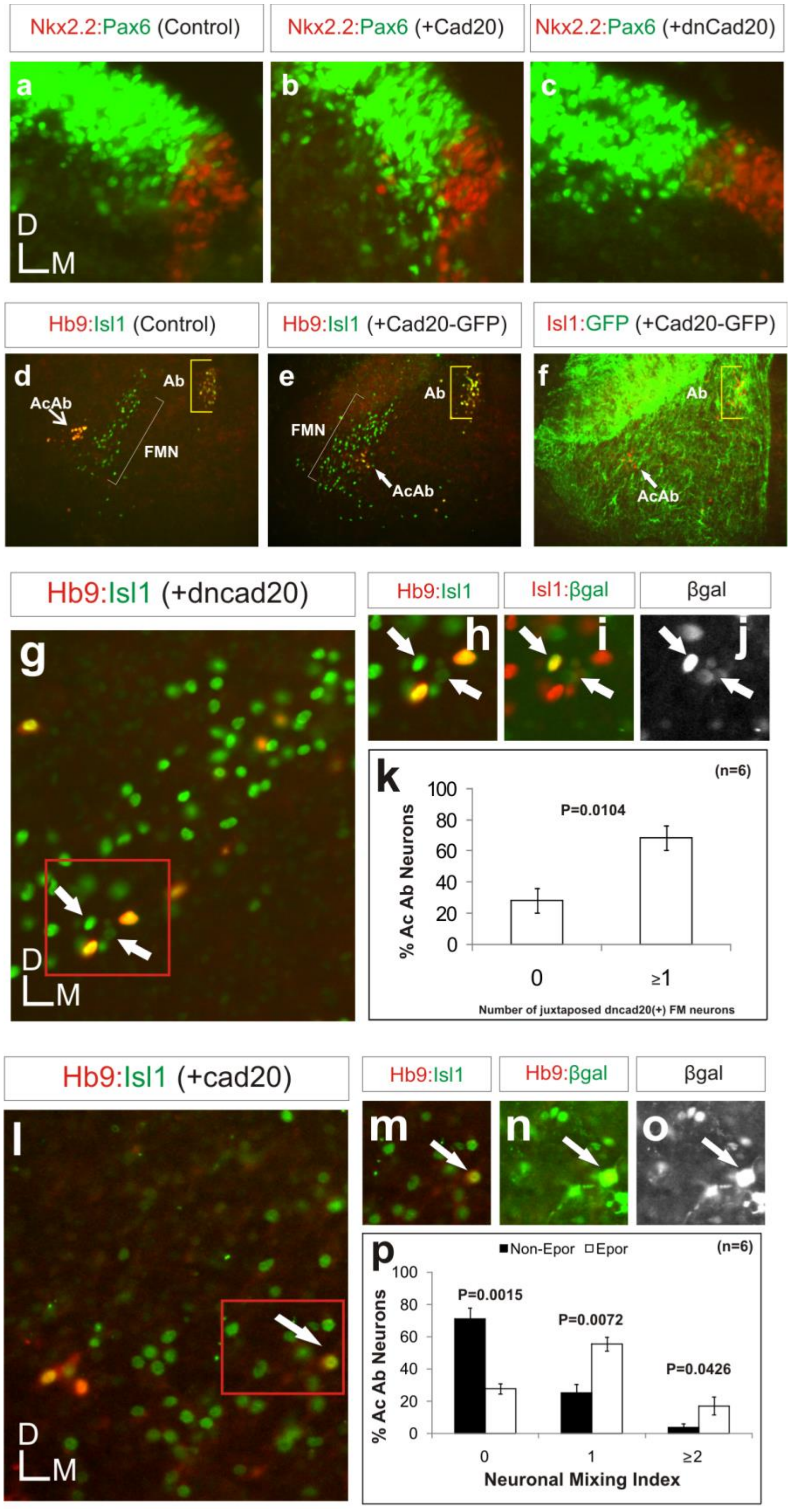
Nkx2.2:Pax6 (Control)
Nkx2.2:Pax6 (+Cad20)
Nkx2.2:Pax6 (+dnCad20)
a
b
c
D
M
Hb9:Isl1 (Control)
Hb9:Isl1 (+Cad20-GFP)
Isl1:GFP (+Cad20-GFP)
d
AcAb
Ab
FMN
e
Ab
FMN
AcAb
f
Ab
AcAb
Hb9:Isl1 (+dncad20)
Hb9:Isl1
Isl1:βgal
βgal
g
h
i
j
D
M
k
(n=6)
P=0.0104
% Ac Ab Neurons
100
80
60
40
20
0
0
≥1
Number of juxtaposed dncad20(+) FM neurons
Hb9:Isl1 (+cad20)
Hb9:Isl1
Hb9:βgal
βgal
l
m
n
o
D
M
p
Non-Epor
Epor
(n=6)
P=0.0015
P=0.0072
P=0.0426
% Ac Ab Neurons
100
80
60
40
20
0
0
1
≥2
Neuronal Mixing Index

Figure S4

Supplemental Figure Legends

Figure S1 related to figure 1. Nucleus segregation, cadherin and catenin expression at rhombomere 8.

Branchiomotor ($Hb9^{-}$/islet-1^{+}) and Somatomotor ($Hb9^{+}$islet-1^{+}) nuclei migrate, comingle and segregate between st20 (a), st26 (b), st29 (c) and st31 (d). This results in the hypoglossal (somatomotor) nucleus taking up a medial and also dorsal subgroup. The vagal nucleus (X) (visceromotor) is dorsal to the dorsal hypoglossal (dXII). The glossopharyngeal (IX) (brachio/visceromotor) nucleus is lateral to all those nuclei. White dotted lines show the midline. e. Costaining of Hb9 immunohistochemistry with *cadherin-6b in situ* hybridisation in r8 at stage 29 reveals that the ventral and dorsal hypoglossal and the glosopharyngeal nuclei all express cadherin-6b.

f. Costaining of Hb9 immunohistochemistry with *cadherin-13 in situ* hybridisation at stage 29 shows that the lateral part of the ventral hypoglossal, the dorsal hypoglossal and the vagal nuclei express cadherin-13.

g. Summary of *cadherin-6b* and *cadherin-13* expression at stage 29.

h. *Islet-1* in situ hybridization at st35 shows the ventral hypoglossal nucleus segregated into two distinct parts, the vagal nucleus and the glossopharyngeal nuclei. Glosopharyngeal nucleus spreads medio-laterally at this stage.

i-o *γ-catenin* (i) *β-catenin* (j), *cadherin-6b* (k), *cadherin-8* (l), *cadherin–20* (m) *cadherin-13* (n) and *cadherin-22* (0) expression by in situ hybridisation in st35 r8 cranial motor nuclei. Note that *γ-catenin* appears expressed in all cranial motor nuclei at r8 whereas *β-catenin* is expressed in only one part of the hypoglossal nucleus.

p. Summary of differential cadherin expression in r8 motor nuclei.

Figure S2 related to figure 2. Developmental profile of cadherins within cranial motor nuclei at r8.

a-c. *cadherin-20* expression in cranial motor neurons marked by islet-1 immunohistochemistry at r8 at st22 (a) and st30 (b). (c) summarises this data; at r8, *cadherin-20* appears in a small subset of motor neurons and this expression apparently persists throughout nucleus segregation. VZ is ventricular zone.

d,e. *cadherin-6b* in situ hybridisation with colabelling of Hb9 immunohistochemistry in r8 at stage 24 (d) and stage 27 (e).

f, g. *cadherin-13* in situ hybridisation with colabelling of Hb9 immunohistochemistry in r8 at stage 24 (f) and stage 27 (g). *Cadherin-13* is expressed in no motor neurons at r8 at either of these stages.

h. Summary of the expression of *cadherin-6b* and *cadherin-13* at r8 at stage 24 and within the dorsal and ventral hypoglossal nuclei at stages 27 and 29.

Figure S3 related to figure 3. Manipulation of cadherin function does not alter brainstem structure or motor neuron migration.

a-c Ventricular zone expression of Pax6 following NΔ390 expression in r5 at stage 29. a. Pax6 expression, b. DAPI staining of cell nuclei. c. Pax6 (red) and GFP (green) marking electroporation with the cadherin dominant negative construct.

d-f Expression of the Progenitor and postmitotic ventral interneuron marker Nkx6.1 following NΔ390 expression in r5 at stage 29. d. Nkx6.1 expression, e. DAPI staining of cell nuclei. f. Nkx6.1 (red) and GFP (Green) marking electroporation with the dominant negative construct.

g. Quantification of motor neuron number in r8 at stage 29 following NΔ390 expression compared to control.

h. GFP labeled motor axons following NΔ390 expression (indicated by arrow). The exit point and initial axonal trajectory is unaffected.

i, j. NΔ390 expression at r5 results in Facial Motor nucleus dissociation in a cell autonomous manner. $Islet^{+}$ /GFP^{+} motor neurons do not coalesce (indicated by arrows), $Islet^{+}$ /GFP^{-} motor neurons do coalesce (compare i with j)

k-m NΔ390-GFP expression disrupts nucleus clustering at r8 as assayed by Hb9 (k, l) and islet-1 (k-m) immunoreactivity. k. shows the control, l,m shows the results of NΔ390 expression marked by GFP immunofluorescence in m.

n-q Quantification of Nucleus Coalescence Index (NCI) of Hypoglossal (n), Vagal (o), Dorsal Hypoglossal (p) and Glosopharyngeal nuclei (q) comparing control to NΔ390 expression.

r-u. Migration of motor neurons is not altered by cadherin function disruption. Membrane RFP expressed under the control of the Hb9 promoter labels entire migrating somatic motor neurons. r. During radial migration MN's are closely associated with tranistin $^{+}$ radial glia (green). s. Motor neuron associations with radial glia are absent during tangential migration (two neurons are shown here). The morphology of radial vs tangentially migrating neurons is different (r, s).

t. Summary of migratory pathway of somatic motor neurons.

u. Quantification of the average position from the VZ of control RFP^{+} vs. $N\Delta390^{+}$ GFP^{+} RFP^{+} somatic motor neurons. Error bars show SEM.

Figure S4 related to figure 4. Cadherin 20-GFP, cadherin 20 and dominant negative cadherin 20 misexpression at r5.

a-c. Nkx2.2 and Pax6 label distinct motor neuron progenitor domains within the Ventricular zone. The structural integrity and gene expression is unaltered following cadherin manipulation; control (a), cadherin-20 misexpression (b), DN cadherin-20 expression(c).

d-f Clustering of Abducens and Facial motor nuclei are not altered following cadherin-20-GFP fusion expression. Hb9 (d, e), islet-1 (d-f) immunofluorescence in st30 control (d) or cadherin-20-GFP electroporated brainstem (e, f) marked by GFP immunofluorescence (green in f).

g-k. Cell autonomous analysis of AcAb/FMN mixing following dominant negative cadherin-20 expression. $Hb9^+$ /$islet^+$ AcAb neurons are mispositioned (g), magnification of boxed region (g), $islet^+\beta$-Gal^+ neurons confirm plasmid construct expression (h-j). Quantification shows that mispositioned AcAb neurons are in contact with ≥1 $islet^+\beta$-Gal^+ Facial Motor Neurons (k).

l-p. Cell autonomous analysis of AcAb/FMN mixing following cadherin 20 misexpression. $Hb9^+$ $islet^+$ AcAb neurons are mispositioned (l), magnification of boxed region (l), $Hb9^+\beta$-Gal^+ neurons confirm plasmid construct expression (m-o). Quantification using NMI showing that $Hb9^+\beta$-Gal^+ mispositioned AcAb neurons exhibit increased aberrant mixing behavior when compared with control ($Hb9^+\beta$-Gal^-) neurons (p). Error bars show SEM.

Supplemental Experimental Procedures

Chick Embryo Preparation

Fertilised Brown Bovan Gold Hen's eggs (Henry Stewart Farms, UK) were incubated in a forced draft incubator at 38°C and staged (as in [14]). All embryos were treated in accordance with the Animals (Scientific Procedures) Act of 1986, UK.

In Situ Hybridisation Histochemistry.

Digoxigenin (DIG)-labelled anti-sense cRNA probes were used for in situ hybridisation histochemistry on 15 μm thick cryostat sections as in [17]. Dual in situ hybridisation with immunohistochemistry was performed by replacing the proteinase K treatment with a 30 minute incubation of the slide sections with 0.1% Triton X-100 in phosphate buffered saline (PBS) (pH7.4). Development of the RNA in situ was performed as normal followed by incubation with the primary antibody for either islet-1 or Hb9 for 16 hours at 4°C. Following this, secondary HRP conjugated goat anti mouse antibodies were applied and ImPact DAB substrate reagent (Vector labs) was used to reveal HRP immunohistochemistry following manufacturers instructions.

Identification of cranial nuclei.

The entire hindbrain was analysed in transverse section with both Hb9 and islet-1 immunofluorescence. Maps of positioning of somatomotor and branchiomotor neurons in the rostro-caudal extent of the brainstem [5] allowed the assignment of rough identity of the cranial motor nuclei that contribute axons to the VIth, VIIth, IXth, Xth and XIIth cranial nerves. Further assignment of nucleus identity was based on maps of transverse sections of the adult chicken

brainstem [10]. Cadherin expression within each nucleus was confirmed for a selected series of cadherins through double immunohistochemistry and in situ hybridization for Hb9 or islet-1 with the cadherin antisense cRNA probe.

Generation of Expression Constructs

Full-length cDNAs for chick cadherin-20, N-cadherin, cadherin-6b, Dominant negative cadherin-20, GFP or NΔ390 were cloned into a pCAGGS or pCAGGS ires nuclear localized β-galactosidase vector. Cadherin-20-GFP was generated by removing the stop codon and cloning the cDNA for eGFP immediately downstream. Dominant negative cadherin-20 (DNcad-20) was generated by removing the cDNA of cadherin-20 from aa 684 to aa 798, which retains part of the juxtamembrane domain. Other constructs used in this work include transposase integrated doxycycline inducible N-cadherinΔ390 [S1-S3] and Hb9:RFP [S4] which was generated and kindly gifted by I. Lieberam.

In Ovo Electroporation

Expression of cDNAs was achieved by *in ovo* electroporation using an ECM830 electro-squareporator (BTX Inc.). ~0.1μl of DNA construct (1-10μg/μl in H_2O with 0.1% Fast Green (Sigma)) was pressure injected into the lumen of the brain stem. Five 30 Volt electrical pulses of 50ms duration evenly spaced over a 5 second period were applied by placing electrodes adjacent to each side of the head of the embryo. Embryos were electroporated at HH stages 12-18 and analyzed at HH stages 25-32. For NΔ390 experiments, doxycycline hyclate (200μl) at a concentration of 0.25 μg/ml in H_20 was applied from stage 20 onwards.

Immunohistochemistry.

Antibodies used in this study were: Rabbit (R) anti-GFP (Invitrogen, 1/1000), R anti-RFP (1/1000), R anti Hb9 (1/5000), Guinea pig (GP) anti-islet1(2) (1/20000), Mouse (M) anti GFP (1/100), R anti β-galactosidase (Abcam, 1/1000) or chicken anti β-galactosidase (abcam, 1/1000), Alkaline phosphatase conjugated Sheep anti-DIG Fab fragments (Roche) (1/5000). The following mouse monoclonal antibodies were purchased from the Developmental Studies Hybridoma Bank, 2D6 and 4D5 (anti islet-1), 5C10 (anti MNR2/Hb9), F55A10 (anti Nkx 6.1), Pax6 (anti Pax6), 74.5A5 (anti Nkx 2.2), EAP3 (anti transitin). Immunocytochemistry was performed essentially as described in [17]. Cryostat sections mounted on superfrost plus glass slides were incubated in PBS for 5 minutes followed by incubation in block solution (PBS with 1% Goat serum (Sigma) and 0.1% Triton-X-100) for 30 minutes at 20°C. This solution was replaced by antibody diluted in block solution and incubated for 12 to 16 hours at 4°C. Following three washes of 5 minutes in PBS, fluorescent conjugated secondary antibodies were incubated with the sections for 30 minutes at 20°C in block solution, washed and mounted with vectashield fluorescent mounting medium (Vector Labs).

Analysis of Radial Migration of Cranial Motor Neurons.

We electroporated a DNA construct that drives Red Fluorescent Protein (RFP) under the control of the murine HB9 promoter [S4]. All RFP positive motor neurons (>100) expressed HB9. We characterized two migratory streams of motor neurons, those closely apposed to transitin expressing radial glia and a second stream that migrated tangentially to the transitin fibres. The morphology of the neurons in each stream differed; those radially migrating

possessing an extended trailing process. We assessed radial distance from the ventricle of the soma of each HB9:RFP expressing motor neuron dependent on whether it was electroporated by the NΔ390 construct or not and which migratory stream the motor neuron was found to be in. The mean distances from the ventricular zone of all four bins of motor neurons were not significantly different from each other. This suggests that the radial migration of motor neurons is not affected by NΔ390 expression for either those neurons migrating along transitin radial glia or those migrating tangentially to the radial glia.

Image Acquisition

Images were acquired on a Nikon Eclipse E80i fluorescence microscope equipped with a Nikon DS5M and Hamammatsu ORCA ER digital camera or on a Leica SPE confocal microscope.

Quantification.

Cell counts were performed across the entire rostrocaudal extent of the nuclei as identified by positive Hb9 and islet 1 staining. Sections were 15μm thick and were cut in series of four; as such each cell count represents approximately ¼ of the total cell number of each nucleus. Cell numbers from the sections of each embryo were collated and each embryo counts as one point in the data set. Mean values were calculated from the total cell number counted for each embryo and standard error was calculated across this data set (n=5 embryos, except where stated). A paired Student's t-Test was performed comparing total cell number counted within each embryo between internal control and experimental hemispheres (contralateral to electroporated side, n=5, except where stated). NΔ390 expression did not alter the total number

of cranial motor neurons at rhombomere 5 (168±58 versus 188±100 (control vs NΔ390 somatic motor neurons), p=0.19 student's t-test; 614±57 versus 584±33 (control vs NΔ390 total motor neurons, p=0.414 student's t-test; n=3 embryos). Additionally, the total number of accessory abducens neurons was unaffected by both cadherin-20 manipulations (42±7 vs 41±4 (control vs cad-20 electroporation), p=0.885 student's t-test; 48±4 vs 44±4 (control vs dominant negative cad-20 electroporation), p=0.60 student's t-test). Similarly, manipulations of cad-6b and N-cad maintained normal neuron number (60±15 versus 51±14 accessory abducens neurons cad6b electroporation, p=0.70 and 46±2 vs 48±13 for N-cad electroporation)

The Neuronal Mixing index (NMI) was calculated essentially as described [17]. In brief, each single neuron of either the accessory abducens or the facial nucleus was scored by the number of neurons of the other nucleus that surrounded it and allocated to an appropriate bin. The percentage of neurons of each type in each bin is reported. Statistical analysis was performed using a student's t-test between experimental and control bins in turn and through a χ^2 analysis of the distribution of neurons across all the bins. For analysis of cell autonomy of cadherin-20 manipulations, each mispositioned neuron was divided dependent upon the presence or absence of GFP/β-galactosidase. NMI was then performed following Cadherin 20 misexpression and a direct comparison made between electroporated and non-electroporated neurons. Following dominant negative cadherin 20 expression, mispositioned neurons were placed in bins based upon their juxtaposition to Islet-1$^+$ Facial Motor neurons; either 0 (GFP/β-gal $^-$/ Islet-1 $^+$) or ≥1 (GFP/β-gal $^+$/ Islet-1 $^+$) . Comparison of the two bins indicated that a mispositioned neuron was likely to be juxtaposed to a facial motor neuron electroporated with the cadherin-20 dominant negative construct.

For the Nucleus Coalescence Index (NCI), individual neurons of the same type were chosen and the number of neurons surrounding it that were of the same identity counted. Neurons in the zero bin are thus separated from their neighbours. Neurons in the remaining bin had at least one neighbour adjacent and were deemed to be within the nucleus proper.

Supplemental References

S1. Kawakami, K., and Noda, T. (2004) Transposition of the Tol2 element, an Ac-like element from the Japanese medaka fish Oryzias latipes, in mouse embryonic stem cells. Genetics *166***,** 895-899.

S2. Sato, Y., *et al*, (2007) Stable integration and conditional expression of electroporated transgenes in chicken embryos. Dev Biol *305***,** 616-624.

S3. Watanabe, T., *et al*, (2007) Tet-on inducible system combined with in ovo electroporation dissects multiple roles of genes in somitogenesis of chicken embryos. Dev Biol. *305***,** 625-636

S4. Wichterle H., Lieberam I., Porter J. A., and Jessell T. M. (2002) Directed differentiation of embryonic stem cells into motor neurons. Cell *110*, 385–397.
